# Supplementary material for: Study protocol for a longitudinal study evaluating the impact of rape on women’s health and their use of health services in South Africa
Source: BMJ Open. 2017 Sep 29;7(9):e017296. doi: 10.1136/bmjopen-2017-017296 (PMC5640088; doi:10.1136/bmjopen-2017-017296)
Supplement: Author's manuscript [file bmjopen-2017-017296.draft_revisions.pdf]

BMJ Open is committed to open peer review. As part of this commitment we make the peer review history of every article we publish publicly available.

When an article is published we post the peer reviewers' comments and the authors' responses online. We also post the versions of the paper that were used during peer review. These are the versions that the peer review comments apply to.

The versions of the paper that follow are the versions that were submitted during the peer review process. They are not the versions of record or the final published versions. They should not be cited or distributed as the published version of this manuscript.

BMJ Open is an open access journal and the full, final, typeset and author-corrected version of record of the manuscript is available on our site with no access controls, subscription charges or pay-per-view fees (http://bmjopen.bmj.com).

If you have any questions on BMJ Open's open peer review process please email
info.bmjopen@bmj.com

BMJ Open

The impact of rape on women's health, use of health services and experiences of violence: a longitudinal study protocol

Journal:	*BMJ Open*
Manuscript ID	bmjopen-2017-017296
Article Type:	Protocol
Date Submitted by the Author:	13-Apr-2017
Complete List of Authors:	Abrahams, Naeemah; South African Medical Research Council, Gender & Health Research Unit<br>Seedat, Soraya; Stellenbosch University, Department of Psychiatry<br>Lombard, Carl; South Africa Medical Research Council, Biostatistics Unit<br>Kengne , AP; South African Medical Research Council,<br>Myers, Bronwyn; South African Medical Research Council, Alcohol, Tobacco and Other Drug Research Unit<br>Sewnath, Alesha; South African Medical Research Council, Gender& Health Research Council<br>Mhlongo, Shibe; South African Medical Research Council, Gender & Health Research Unit<br>Ramjee, Gita; HIV Prevention Unit, Medical Research Council; HIV Prevention Unit, Medical Research Council<br>Peer, Nasheeta; South African Medical Research Council, Non-Communicable Disease Research Unit<br>Garcia-Moreno, Claudia; World Health Organisation, Department of Reproductive Health and Research<br>Jewkes, Rachel; South African Medical Research Council, Gender & Health Research Unit
**Primary Subject Heading**:	Public health
Secondary Subject Heading:	HIV/AIDS
Keywords:	Rape, MENTAL HEALTH, Gender Based Violence

SCHOLARONE™
Manuscripts

Title: The impact of rape on women's health, use of health services and experiences of violence: a longitudinal study protocol

Authors:
Naeemah Abrahams: (corresponding author)
Gender & Health Research Unit
South African Medical Research Council
P O Box 19070 Tygerberg
Cape Town, 7405
Tel: 021 9380445
Fax: 021 9380310
Email: nabraham@mrc.ac.za

Soraya Seedat: Anxiety and Stress Disorder Unit
University of Stellenbosch
Cape Town, South Africa
Tel: +27 21 9389161: Email:sseedat@sun.ac.za

Carl Lombard: Biostatistics Unit
South African Medical Research Council
Cape Town, South Africa
Tel:+27 21 9380328: Email: carl.lombard@mrc.ac.za

Andre Kengne: Non-Communicable Disease Research Unit
South African Medical Research Council
Cape Town, South Africa
Tel: +27 21 9380841: Email: Andre.Kengne@mrc.ac.za

Bronwyn Myers: Alcohol, Tobacco and Other Drug Research Unit
South African Medical Research Council
Cape Town, South Africa
Tel: +27 21 9380993: Email: bmyers@mrc.ac.za

Alesha Sewnath: Gender & Health Research Unit
Durban, South Africa
Tel: +27 31 2423721: Email: Alesha.Sewnath@mrc.ac.za

Shibe Mhlongo: Gender & Health Research Unit
Cape Town, South Africa
Tel: +27 31 9380826: Email: Shibe.Mhlongo@mrc.ac.za

Gita Ramjee: HIV Prevention Research Unit
Durban, South Africa
Tel: +27 31 2423635: Email: gita.ramjee@mrc.ac.za

Nasheeta Peer: Non-Communicable Disease Research Unit
Durban, South Africa
Tel: 27 31 2034882 : E-mail: nasheeta.peer@mrc.ac.za

Claudia Garcia-Moreno: Department of Reproductive Health and Research, World Health Organization

For peer review only

Geneva, Switzerland
Tel: +41 22 791 4353: Email: garciamorenoc@who.int

Prof Rachel Jewkes: Gender & Health Research Unit
Pretoria, South Africa
Tel: 0123398525: Email: rjewkes@mrc.ac.za

Word Count: 5953

For peer review only

Introduction

In South Africa between 12-28% of adult women have been raped and 29% have HIV. Although the interface between gender-based violence, HIV and mental health has been described, there are substantial gaps in knowledge of the medium- and long- term health impact of gender-based violence. The 2010 Global Burden of Disease excluded many health outcomes associated with rape and other forms of gender-based violence as the systematic reviews revealed huge gaps in the data and the poor evidence of health effects. This study aims to describe the incidence and attributable burden of physical (including HIV acquisition) and mental health problems in adult women over two years post-rape, through comparison with a cohort of women who have not been raped.

Methods and Analysis

This longitudinal study seeks to recruit 1008 rape exposed and 1008 rape non-exposed women. Women will be recruited from health services and assessments will be at baseline, 3, 6, 9, 12, 18 and 24 months. Outcome measures include exposure to risk factors, mental health status, cardio-metabolic risks, biomarkers for HIV, STIs, pregnancy and stress. The primary analysis will be to compare HIV incidence in the two groups using logrank tests and appropriate models to predict health outcomes overtime will also be applied.

Ethics and Dissemination

With rape a key element of the study, the safety and protection of participants guides the research process. Although the research involves minimal risk to participants, questions may be intrusive and trigger emotions leading to short term psychological distress. Support forms a critical aspect of the study with a trauma counsellor part of the research team. The study will substantially advance our understanding of the impact of rape and will generate robust data that will assist in the development of post-rape health services and delivery of evidence-based care for survivors of rape.

Keywords: Rape, HIV, health impact

For peer review only

Strengths and Limitations of the study

- The study will build knowledge in an area with many gaps and contribute to describing the health burden of gender based violence
- The study uses a longitudinal design with a large sample
- The study combines biological and behavioural data
- Study conducted in a developing setting

For peer review only

BACKGROUND

Lifetime exposure to rape and other forms of sexual violence is relatively high in most countries, as reported in global prevalence studies released in June 2013.[1 2] Although aspects of the health impact of rape have been described, the data available has major limitations. Systematic reviews to establish the magnitude of health effects associated with exposure to physical and sexual intimate partner violence (IPV) and non-partner sexual violence including rape, indicate that current evidence is overwhelmingly based on cross-sectional studies and/or clinical populations.[2] Further, the conditions studied are mostly limited to mental ill-health and aspects of reproductive health. It is thus difficult to quantify the health burden in the absence of high quality outcome data from longitudinal studies.

Prevention of HIV acquisition post-rape has been one of the main targets of health care following sexual assault.[3] However, the pathways to HIV acquisition and health outcomes post-rape have yet to be fully elucidated. While HIV can be acquired during the rape event, rape may indirectly elevate HIV acquisition risk in the medium- and long- term through other possible pathways. Evidence for these pathways stems from research on IPV and HIV.[4] One pathway is via acquiescence where exposure to IPV reduces women's ability to assert themselves in sexual contexts and determine the timing and circumstances of sex, including condom use and the insistence on fidelity.[5 6] The second pathway is through elevated high risk sexual behaviour on the women's part, including engagement in sex work, transactional sex, seeking older partners and having multiple partners.[6-8] The third pathway follows the idea that women themselves may not change their behaviour but may be drawn to or sustain relationships with men who are at higher risk of being HIV+ than other men. There is evidence that men who use violence towards women, including rape, are riskier, have more partners, have more sexually transmitted infections (STIs) and are more likely to have HIV infection, as are men who are more likely to partner more vulnerable women.[9-12]

It is likely that all of these pathways are important post-rape and that psychological reactions to rape and rape stigma are powerful mediators of these pathways. These are, in turn, influenced by the circumstances of the rape and there is some evidence that self-blame results in greater and more enduring psychological impact and poorer post-traumatic stress disorder (PTSD) treatment response[13 14]. Evidence from rape counselling services suggests that court processes after rape may be particularly stressful for women,[15] but health impact in general has not been empirically described. Research is needed to test the pathways described above in relation to rape exposure to enable a better understanding of their relative importance, which is critical for developing tailored HIV prevention interventions. Further, there is a need for research that combines clinical assessments of mental health (i.e. PTSD and depression) post-rape with biological markers of stress. The prolonged release of cortisol in the body has been linked to several psychiatric disorders, including PTSD,[16] and it has been suggested that this itself may impact immune system functioning and create enhanced susceptibility to HIV infection. Further research is needed to investigate links between rape, mental ill-health and dysfunctional hypothalamic-pituitary-adrenal (HPA) axis activity.[17 18]

Evolving research is contributing to the better understanding of the role of genetics in psychiatric disorders including responses to trauma such as sexual violence. Psychiatric genetics has the potential to inform our understanding of neuropsychiatric disorders[19] including the development of PTSD and other mental health outcomes post-rape, and to understand more about why not all survivors will develop mental health disorders[20 21]. Factors other than the traumatic event are likely to contribute to their development. Environmental influences, such as trauma (rape), can trigger epigenetic changes resulting in genes being silenced or expressed.[22] Epigenetic mechanisms can therefore be a mediator between genes and the environment. Research is needed to deepen our knowledge on the role of epigenetics in the development of mental health disorders post-rape.

For peer review only

The relationship between non-communicable diseases (such as cardio-metabolic diseases) and rape has not been described in longitudinal studies in the literature. Suggestions of a potential relationship can be gained from cross-sectional research on the associations between physical and psychological IPV and self-reported chronic diseases including hypertension, diabetes, asthma, heart disease, cancer and arthritis.[23] It has been suggested that the stress response following rape may lead to the development of chronic diseases and its risk factors via similar causal pathways as in individuals exposed to general psychological stress which are known mediators of poor health care[24]. Direct pathways have been hypothesized via physiological mechanisms when traumatic stressors activate a cascade of physiological responses that affect the cardiovascular, metabolic, neural, endocrine, and immune systems.[24-26] These include pathways via behavioural coping mechanisms such as detrimental lifestyle choices. Research is needed to understand these associations and the potential pathways using biological markers. Many studies, using different study designs and approaches, have examined the relationship between psychosocial stress and cardiovascular disease (CVD) and its risk factors.[27-29] None of these have described the relationship between psychosocial stress triggered by rape exposure and the development of CVD risk factors in longitudinal studies.

In sub-Saharan Africa, with a high population prevalence of HIV infection, it is common for women who are HIV positive to be raped. Not much is known about HIV positive women who are raped and the specific health burden associated with such exposure. In recent years there has been a substantial increase in HIV testing across sub-Saharan Africa, especially among women attending antenatal services and much greater linkage to HIV treatment services.[30 31] High levels of medication adherence and retention in treatment are essential for the effectiveness of anti-retroviral therapy in preventing HIV disease progression and mortality. Consequently, research on barriers to adherence and retention in HIV treatment and care services has been increasing.[32] Psycho-social factors are one group of factors that undermine linkage and retention in care, and among rape survivors, mental illness and stigma are of particular concern because they are recognised barriers to HIV adherence and retention in care.[33 34] Understanding and developing interventions to respond appropriately to the impact of rape on women's ability to adhere to treatment and remain in care is important for optimising women's HIV treatment outcomes.

In order to fill these gaps in knowledge we are undertaking research that has the overall goal of advancing the understanding of the health consequences of rape, notably HIV and mental health. This study is particularly well suited to being conducted in South Africa as it is a country with high sexual violence and HIV incidence[9 35] and the country with the largest population of individuals living with HIV.[36]

METHODS/DESIGN

Research Aims

The study has one primary aim and eight secondary aims.

Primary Aim

To determine the incidence and attributable burden of HIV acquisition in adult women up to 24 months post-rape, in comparison to those of a cohort of women who have not been raped.

Secondary Aims

1. To determine the incidence, attributable burden and recovery rates of physical and mental health problems that may enhance HIV risk at 3, 6, 9 ,12, 18 and 24 months.
2. To determine the individual, relational, social and criminal justice risk factors for health problems at the different time points that may be HIV risk factors and associated with the persistence of symptoms.
3. To estimate the relative importance of the different hypothesised pathways to HIV acquisition.

For peer review only

4. To determine the impact of rape on HIV positive survivors' ability to link to HIV care, retention in treatment and on their sexual risk taking behaviour.
5. To evaluate changes in cortisol levels and other steroid hormones (cortisone, testosterone, progesterone, corticosterone, and DHEA) over time measuring levels in hair, and to evaluate the stress-response of women before and after the traumatic event of rape.
6. To investigate genetic and epigenetic factors as participating biomarkers in the etiology and trajectory of PTSD among rape-exposed women.
7. To determine the incidence and attributable burden of CVD risk factors, markers of increased CVD risk, and hypertension and diabetes related renal dysfunction comparing rape-exposed and non-exposed women.
8. To explore, qualitatively, the experiences of both rape-exposed and rape non-exposed women and to focus on motivations and experiences of the research process, retention issues and the support provided to them.

This comparative cohort study includes a 24-month follow-up. The sample size calculation addresses the primary aim. This is based on a log-rank test comparing the HIV incidence between rape exposed and non-exposed groups. To achieve 90% power to detect a significant difference in HIV incidence, assuming an annual incidence of 9% and 6% among the rape exposed and non-exposed groups respectively, and given an expected 36 month accrual and a minimum follow-up of 12 months, at a 5% significance level and 10% dropout, 193 HIV+ incident cases will be required. A minimum HIV negative cohort of 780 participants is required in each of the exposure groups. We will recruit at least 844 HIV negative participants in each exposure group and the last participant recruited will be followed up for 12 months. Research in Kwa-Zulu Natal (KZN), where this study is being undertaken, has found an average annual incidence of 10% among young women participating in trials.[37] We anticipate that 20% of participants will be HIV+ at baseline and, as such, a total sample size of 2016 (1008 per arm) will be recruited. We anticipate a 10% drop-out based on previous experiences with cohorts in KZN where 95-97% retention was achieved in five longitudinal studies.[38] All participants will have data collected at baseline 3, 6, 9 and 12 months follow-up. Participants recruited earlier in the study will also have data collected post 12 months, at 6-monthly intervals up to 24 months, which will give greater power to the study.

Setting

The study participants are recruited from rape services in the province of KZN, including the Thuthuzela Care Centres (TCCs), which are managed by the National Prosecuting Authority, and other hospital-based Crisis Centres. Never-rape exposed participants are recruited from family planning clinics. The TCCs are one-stop centres based at public hospitals providing 24 hour integrated care to rape survivors including access to police, counselling and medical care.[39] Permission to recruit at the TCC was granted by the National Prosecuting Authority and the Provincial Department of Health granted permission to recruit at family planning clinics. Our research site is within a hospital complex with easy access to public transport.

Inclusion and exclusion criteria

Rape exposed participants are women aged 16–40 years, who have lodged a complaint of rape within the previous 20 days at a rape centre. We exclude children under 16 years as they constitute a vulnerable group requiring additional ethical safeguards. The upper age limit of 40 has been chosen as the HIV incidence in women is much lower over age 40. The limit of 20 days has been chosen to ensure that time points for data collection are reasonably standardised across the study population and to allow for the documentation of acute stress reactions and HIV status at baseline (versus PTSD which will be captured at the follow up visits). Participants are excluded if they are very severely mentally distressed, mentally disabled or more than 14 weeks pregnant. Participants attending family planning (non-rape exposed) are matched for age (+/- 1 year) with recruited raped-

For peer review only

exposed women. The choice of family planning clinic clients provides a sexually active control population who, like the women reporting having been raped, are also engaged with services. Women who report lifetime exposure to sexual violence, as determined by a screening questionnaire, are excluded from the non-exposure cohort. Other exclusion criteria, as for rape-exposed women, are applied. We are following the model of recruitment that has previously been successful in studies at sexual assault services in South Africa. These include a series of information giving and consent processes described below and also described in detail elsewhere.[40 41]

Assessment of exposure and outcomes measures

The interview content and outcome measures are listed in Table 1. Baseline interviews are conducted face-to-face in the language that participants are comfortable with (Zulu, English). Responses to questions at follow-up interviews are interviewer administered or self-completed depending on participant literacy levels and familiarity with computers/tablets.

Table 1: Exposure and outcome measures at each data collection time point

	Variable group	Measures	Source of measures
A	IPV exposure	Ever physical, sexual, emotional<br>Past year, post-rape	WHO multi-country study instrument[42][tested in South Africa by Jewkes et al 2006 [43]
B	Rape circumstances and physical trauma / injuries	Rape circumstances: # perpetrators, relationship to perpetrator, weapon use, degree of violence, type of rape<br>Intimidation by the perpetrator or friends.	From Jewkes et al 2010,[44](*questions to be asked after criminal case have been closed)*
C	Rape stigma and social support	Rape stigma and self-blame<br>Coping mechanisms<br>Rape disclosure<br>Social support<br>Social disruption – leaving home, school, relationship	Social Support Appraisal Scale;[45]<br>Brief COPE;[46]<br>South African Stigma Scale[47]
D	Demographic factors	Demographic factors: age, income, education, employment, Relationship status, sexual orientation	From Jewkes et al 2006[43]
E	Mental health outcomes	Depression<br>Anxiety disorders<br>PTSD<br>Suicidality<br>Other Anxiety disorders<br>Alcohol & drug abuse<br>Cortisol level in hair<br>Epigenetic changes<br>Telomere length	MINI for psychosis and anxiety disorders;[48] CES-D (depression);[49] AUDIT (alcohol);[50] DUDIT (drugs);[50-52]<br>Life Events Checklist (LEC);[53]<br>Davidson Trauma Scale (DTS for PTSD)[54 55]<br>Cortisol in hair and Epigenetics analysis from blood samples
F	Other trauma	Previous trauma: child abuse, other interpersonal violence or traumatic life events<br>*Follow up interview: rape, interpersonal violence or traumatic life events since the last interview*	Childhood trauma scale Bernstein et al[56] modified for South Africa Jewkes et al;[57]<br>Life Events Checklist; Rape and forced sex measures from Jewkes et al 2006;[43]
G	Criminal justice processes and circumstances of the rape	Case progression: case dropped, arrest, bail, guilty plea, case withdrawn, trial commenced, testimony in court given, trial outcome<br>Perceived satisfaction with the system including information given about whereabouts of accused	Jewkes et al 2010;[44] satisfaction questions to be developed;<br>*(These questions will be asked once criminal case have been closed)*

For peer review only - http://bmjopen.bmj.com/site/about/guidelines.xhtml

H	Acquiescence to male domination of sex	Attitudes to gender relations and perceived social norms<br>Consistent condom use<br>Relationship control scale<br>Sexual frequency	from Jewkes et al 2006;[43] attitudes developed in South Africa and validated in Jewkes et al 2003;[58]
I	Own sexual risk behavior	Transactional sex<br>Partner numbers ever & past year<br>Concurrency	from Jewkes et al 2006;[43] 2010;[57]
J	Partner riskiness	STIs of current partner<br>Use of IPV by current partner<br>Partners concurrency /polygamy	from Jewkes et al 2006;[43] 2010;[4]
K	Biological risk enhancers or co-factors	Hormonal contraception use at baseline and on-going<br>Pregnancy loss (*since last interview)*: termination, miscarriage	Questions from the Demographic and Health Survey[59]
L	Cardio-metabolic risk assessment	Screening questions for risk assessment.<br>Measurements: hip and waist, BP, BMI, glucose status (glycaemia/Hba1c) and lipid panels<br>CRP ultrasensitive (cardiac), Gamma-GT, ALT(SGPT), AST(SGOT), Creatinine (serum),	Questions from the WHO STEPs questionnaire[60]
M	Health-care seeking practices post-rape health, HIV testing and adherence	Completion of PEP & STI treatment<br>Emergency contraception,<br>HIV testing post-rape & previously<br>HIV disclosure<br>Attendance for health assessment<br>Commencement of ART & adherence<br>Experience of HIV-related ill health<br>Satisfaction with health care provision	Items developed and tested in pilot
O	Reproductive and Child Health	Contraception, Fertility, Pregnancies, reproductive health,<br>Child health including babies born during RICE study participation	Questions developed and adapted from DHS

Data is being collected in real time using an electronic data management system. Trained staff administer the mental health diagnostic assessment face to face, the MINI DSM-IV, diagnostic interview.[48] The Research Nurse does the clinical assessments, collects the biological specimens (Table 2), conducts HIV testing and counselling, a pregnancy test and an STI test (self-administered vulval swab for Trichomonas, which is treated if positive). We follow the WHO recommendations for HIV rapid testing by doing a second test on all positives as well as an ELISA test (Vironostika HIV1/2 Elisa).[61] Standard pre-and post-test counselling is delivered in a culturally-sensitive manner. Specimens for HSV2 testing are done at 6-monthly intervals from those who are HSV2 negative at baseline. CD4 count and viral load are measured 6-monthly as is the routine in the public sector clinics, so as not to overly influence health-seeking behaviour by observation.

Table 2 Clinical assessments and biological samples at each visit

	Baseline	3 Months	6 Months	9 Months	12 Months	18 Months	24 Months
Biometrics	X	X	X	X	X	X	X
Clinical assessment	X	X	X	X	X	X	X
Mental health assessment	X	X	X	X	X	X	X
HIV and trauma counselling	X	X	X	X	X	X	X

For peer review only

Laboratory evaluations							
HIV rapid test and ELISA	X	X	X	X	X	X	X
Pregnancy test	X	X	X	X	X	X	X
Trichomonas swab	X	X	X	X	X	X	X
HSV2	X	X	X	X	X	X	X
Glucose/lipid panels CRP ultrasensitive (cardiac), Gamma-GT, ALT(SGPT), AST(SGOT), Creatinine,	X			X			X
Cortisol (*Hair Sample*)	X	X	X	X	X		
Genetic & epigenetic tests	X	X	X	X	X	X	X
VIROLOGY (HIV+)							
Viral Load & CD4	X		X		X		X
p-antigen 24 (*Sero-converted)*	X	X	X	X	X	X	X
Storage							
Plasma	X	X	X	X	X	X	X
Serum	X	X	X	X	X	X	X

Follow-up interviews and retention

Participants are given a return date and telephonic contact is maintained between interviews. The study team understands that rape survivors' lives are often in flux in the immediate period after rape and this can lead to considerable mobility and a change in contact details. Although the study retention team (retention counsellor, data manager, research assistants responsible for home visits) are primarily responsible for problem solving retention barriers, all RICE staff are cognisant that their interactions with participants can influence retention. A tracking system to monitor retention has been developed and is updated daily and this allows for immediate intervention (see discussion below on retention challenges).

Data management and analysis

We use a biometric system to identify, track and link participants and their different data sources (i.e. online interviews, clinical data, and biological samples) across visits. A unique barcoded participating identification(PID) number is generated at enrolment and we use barcodes on all samples and documents. Data is collected and entered in real-time on a dedicated website and monitored daily by the data manager. Data is downloaded to a secure server and kept in a password protected system. Laboratory results are merged into the main dataset using the unique PID. The primary analysis will be to compare HIV incidence in the two groups. The logrank test will be used to compare the groups taking into account the interval censoring of event times that will occur due to the visit schedules. The Wilcoxon-type test will be used. To quantify the risk of infection a Cox proportional hazards model will be fitted and the hazard ratio and 95% confidence interval estimated. Interval censoring will also be taken into account in this inference. Mixed effects regression models will be used to model the mental health status of participants over time and for the comparison of the mean profiles between exposure groups. A random effects model for participants will be used to account for the clustering effect of the repeated measures. Mixed effects models restricted to the rape exposed group will be used to evaluate the association of the individual, relational, social and criminal justice factors on mental health and similar analyses will be done for different groups of interest i.e. HIV positive group. We will also examine a cascade model

For peer review only

depicting the different pathways with the cohort of women who are HIV+ and have been raped and compare it to HIV+ who have not been raped. We will handle missing data by using multiple imputation methods but will not impute for key outcomes.

Ethics

Ethical approval for the study was granted by the South African Medical Research Council's Ethics Committee. Rape is a key element of our study and the safety of participants is paramount and guides the research process. Project staff received extensive training on the ethics, safety and protection of participants. We use lessons learned from previous studies of participants recruited at rape centres.[40 41] We introduce the study to participants as a *Women's Health and Wellbeing Study* to protect women from being identified as rape survivors. The study follows the WHO safety guidelines on doing research on gender-based violence and applies good clinical practice.[62] We provide women with sufficient information as required by the Ethics committee on specific areas such as biometrics, children involvement (see below) genetic samples, HIV testing, hair samples and storage of bloods. There is a separate consenting process for each of the above. We developed a brochure and information sheets for participants. Our previous experience highlighted the importance of providing full information, time to reflect, and testing the understanding of consent before consent forms are signed. Participants are given a clear explanation of voluntariness of participation and the option of withdrawal is given at each visit.

Our study includes 16 and 17 year old girls who are recruited in crisis centres in hospitals and for each we seek consent from both the parent/legal guardian and the child. It is important that the children we approach understand that their attendance at the rape centre will be revealed to the parent/legal guardian if disclosure has not yet occurred. We are cognisant that some children may not want their parent/guardians to know about the rape event and we will not recruit children if they are opposed to disclosure. The same apply to children recruited at the Family Planning clinics. We consider children of 16 years to have sufficient maturity and mental capacity to understand the study (this is acknowledged in the Children's Act No 38 of 2005 as amended by Act 41 of 2007), and the information sheet for children is not different to those we have developed for adult women. We only enrol a child if both the parent/legal guardian and the child give consent to participate. We do not seek consent for HIV testing from the parent/legal guardian as according to South African law, parental consent is not required.

The research involves minimal risk to participants. However, questions may be intrusive and trigger emotions which can cause short term psychological distress. All staff are trained to respond to any distress during the interviews and to provide containment. A trauma counsellor, employed at the start of the study, is available to provide support to participants and to staff. Women who require further assistance are referred to appropriate services. The National Prosecuting Authority trained the staff on what may/may not be discussed with rape survivors to ensure the research does not compromise the legal process. To protect the project staff from having to testify in a trial and the integrity of the legal process, the questionnaire does not ask about circumstances of the rape until the rape case has been closed, or the trial has commenced.

We do not expect any major medical risks as part of the study. Participants may find the prospect of an HIV test distressing but we anticipate that most participants would have been tested for HIV (from the rape care received, antenatal care or family planning services) and will know their status. In addition, there have been major national HIV testing campaigns across the country.[63] Our research nurses are trained to do HIV counselling and, together with the study trauma counsellor, can provide appropriate support and referral.

For peer review only

Project staff monitor adverse events at each visit. A serious adverse event is defined as experiencing of violence by a partner due to participation in study, injury or hospitalization due to the violence, or death of study participant for any reason. The research nurse monitors and documents the occurrence of adverse events (since the last study visit) at each visit and will follow appropriate procedures for notification and response to adverse events that arise. The number of events occurring in each cohort will be enumerated in order to determine whether there is a differential risk between study arms. The data manager will also monitor the adverse event data. Project staff are responsible for determining whether participation in the study is related to the harmful event or not by exploring the circumstances surrounding the event. All events are reported to the SAMRC Ethics Committee.

All staff are trained on confidentiality. All information is stored on secure computers or servers and protected by firewall and passwords. Study data are kept separate from the forms that can link data to the participants (consent forms and retention forms). Participants are compensated with R80.00 (US$ 5.5) at the baseline interview with an incremental addition of R20 (US$ 1.3) on each follow-up visit. Participants are compensated for travel costs or transport is arranged if required. During the site visit participants are offered snacks such as a sandwich and a cool-drink as well a choice of a small chocolate or a packets of peanuts.

STUDY PROGRESS AND CHALLENGES

A pilot study to assess the feasibility of recruitment and administering the assessment schedule was conducted between June and October 2014. We have had a few changes to the initially protocol, mainly the change of recruitment window period and the addition of study sites, children and sub studies (addition of genetics, epigenetics, cardiovascular samples and the qualitative component). Recruitment and enrolment commenced in November 2014. By February 2017, we had achieved 52.8% of the recruitment target for the rape cohort (n=532) and slighter better rate among the non-exposed cohort (58% n=582). Our recruitment rate per month among the rape exposed cohort improved from 13 per month in 2015 to 23 per month in 2016 and this was mainly due to interventions we discuss below. We expect this to improve slightly more and hope to have our full rape cohort enrolled by August 2018. The expected end date of the study is August 2019 when the 12-month interviews of the last participants will be done.

Recruitment and enrollment challenges

Our recruitment is much slower than what we anticipated in the planning phase. This is for various reasons. Our initial plan was to enroll participants within 5 days of the rape event and we found this a huge challenge and enrollment was very slow. The window period for enrollment was extended to 10 days and later to 20 days post-rape. We cannot enroll beyond 20 days post-rape as we assess mental health outcomes such as PTSD, which is sensitive to the timing of the traumatic event and HIV status at baseline is a key measurement. We also found that a larger proportion of patients reporting rape to the TCCs are children, which is different to the age profile statistics provided by the National Prosecuting Authority (NPA) at the planning phase of the study (we used 2011-2013 NPA data to plan enrolment strategy). We also do not recruit mentally challenged survivors or those who are too emotional to discuss research participation. Our initial recruitment process was dependent on the trained TCC staff to identify eligible participants, introduce the study, gain consent to be contacted by us (the researchers) and finally collect accurate contact details. The RICE staff then contacted the potential participant and invited them to our study clinic for formal consent procedures and the baseline interview. Although TCC staff was keen to assist, our study was not their priority; therefore, we enlisted the Life Line counselling staff who provide the initial counselling service at the rape centres and contributed towards their salary. However, when funding (USAID funding) for counselling services at the rape centers decreased at the end of 2015, the RICE investigators decided to employ dedicated RICE recruiters as recruitment remained low and it made

For peer review only

financial sense to employ staff to increase the enrollment rather than enroll over a longer period. We employed two of the Life Line counselors to cover four recruitment sites. They set up the initial appointments, and arrange the collection and transportation of the participants to our research clinic. Frequently, participants become non-contactable after agreeing to attend our research site for formal consent procedures and this is a challenge. Participants provide incorrect contact numbers or our calls remain unanswered (voicemail), the latter often happens when final arrangements are done or at time of transport pick-up. Such behavior is also noted among the non-exposed group recruited at family planning clinics and we suspect potential participants are reluctant to inform us directly that they do not want to participate in the research. They may feel socially coerced to agree but find a way to discontinue contact with us. We know that rape survivors' lives are difficult post-rape and they change their phone numbers (phones are sometimes lost/stolen at the time of the rape) or they relocate. We also know that their mental health status interferes in their ability to regain normality.[13 64] This is an inherent challenge in our study and staff is sensitive to rape survivors and accept that this group of women may not want to participant in our study.

Retention challenges and responses

Retaining participants in longitudinal studies is challenging and rape survivors bring additional concerns. We searched for lessons learned from other studies but could not find longitudinal studies with large rape cohorts- although the HIV/AIDS sector have always grappled with retention in cohort studies.[65 66] As indicated above we have participants who relocated including to other provinces for safety reasons or as a coping strategy or they found jobs in other towns. We are aware survivors experience a range of emotional difficulties with varying levels of distress and coping skills, and we know receiving support and care from others assist them on their road to recovery. We also know that there is poor integration of mental health within rape services and although women can return to the rape centre they often choose not to. Our study has a full-time qualified trauma counsellor who provides counselling support at the study site; however, many do not use this facility and we may need to understand this better in follow-up studies. We find that women from both cohorts who are counselled at our clinic often seek assistance for their daily struggles. Women will report their stress are related to their difficult lives, such as trying to find a job and securing income, and counselling sessions are often about assisting them in coping with these hardships. We expected more women to withdraw from the study because the research reminded them of the incident, but among the 26 women who withdrew only two mentioned this as the reason. There may be others who are withdrawals but because this has not yet been communicated to us we consider them loss to follow up (LTFU).

We have a number of activities to support retention beyond reimbursement such as collection of good locator information, which is updated at each visit (including consent to use social media), maintaining telephone contacts between clinic visits, individual birthday wishes via WhatsApp, offering transport from the rape centre, and a Saturday morning clinic to accommodate students and working women. We also try to offer women a pleasant experience at the clinic and offer manicures and nail painting. We constantly exploring other ways to improve experiences and currently exploring offering head and neck massages (10-15 minutes). At the end of 2015, our retention was between 50-75% (depending on the visits) and we intensified our retention activities with an incremental increase in reimbursement monies for each additional visit, a present (necklace/bag/earrings) for those who attend their 12- and 24-month visit and an additional snack of a small chocolate or a packet of peanuts. We employed a retention counsellor whose primary task is to track and monitor participant attendance and to do problem solving counselling of retention barriers identified. We also employed a driver for home visits, initially for once a week but increased this to twice a week. In Jan 2017, we employed two drivers, one dedicated to daily home visits to those who missed a visit, and the other for the collection of participants. However, home visits are also not always successful. Sometime a home is never found or the participant is not known by the

For peer review only - http://bmjopen.bmj.com/site/about/guidelines.xhtml

household; this may be deliberate mis-information given to us as a form of avoidance. We also find homes where participants have left after renting at time of recruitment and contact is completely lost. However, many home visits result in finding participants or getting updated telephone numbers.

We developed an early warning system of missed attendance and home visits are done soon after a missed appointment. These retention and tracking activities have become more complex as the number of baseline participants and follow-up visits increase. Each of our 2016 participants are expected to attend seven times (14 056 clinic visits). We also started a monthly raffle where a grocery or pamper parcel worth R150.00 (US$10) can be won. Participants enter their study number into the visit box at each visit and a draw is made at the end of each month. Transport to the clinic remains one of the main challenges. We recruit women from a wide geographic area and our study site in Chatsworth is only accessible by local taxi services, which is very unpredictable. Our participants report spending a long time travelling. Since the start of 2017, we offer all women transport to the clinic and our driver collects participants in the morning at their homes, the rape centre or a known agreed landmark (police station/taxi rank etc.). Some women do not use the collection service as they profit slightly from the transport reimbursement and therefore prefer to use public transport. Such coordination requires very good communication among staff members, and between staff and participants, which is possible using WhatsApp - especially after hours when arrangements are finalised.

DISCUSSION

We have learned many lessons on how to setup and manage a longitudinal study with rape victims and we continue to learn as our study progresses. We do not expect enrollment of rape-exposed participants to increase much beyond 26 per month as we have few other options left to increase recruitment from these four sites. Broadening the recruitment sites is not financially feasible. We have to accept that some women are not assertive enough to tell us that they do not want to participate in our study and to avoid disapproval they agree initially and thereafter evade contact. We also have to be cognisant that power imbalances between researcher and participant may also be at play. More importantly the period post-rape is very difficult for survivors and they have to negotiate many unsympathetic environments which may include the rape services (health, police, justice, social), unsupportive family and friends as well as a broader social environment wherein rape stigma remains a reality.[13 40]

Loss to follow-up (LTFU) is the most common cause of attrition in cohort studies[65] and the loss of contact with the participants has caused frustration among staff. Continuous support and allowing staff to discuss their frustrations has enabled them to continue interacting sympathetically with trauma victims and to prevent vicarious trauma. The RICE study counsellor supports staff in addition to participants. We also ensure staff have access to an expert psychologist who is available to them and spends a day with them every quarter to assist them in working with traumatized rape survivors. Staff often said they are encouraged when returning participants are clearly on a healing path (i.e. less depressed or acceptance of a HIV status and starting ART) which makes the work a rewarding experience.

Difficulties in retention is well known in cohort studies. We have learned much and in hindsight, we should have employed the retention counsellor earlier in the study and should have placed a greater effort on the home visit programme. We are continuously assessing and discussing how to improve retention and we hope the two recent initiatives (transport service and the raffle) will attract people to return. We may not be aware of all the factors influencing retention and we hope the data from home visits and from our qualitative study will provide some insight to assist us in future longitudinal studies with sensitive participants. This study will also allow us to explore differential retention and

For peer review only

loss to follow-up between the two groups. Mental health may also influence retention of women who are depressed and/or anxious and/or using substances and reluctant to attend the study clinic. Well described in the literature on rape research is the problem that women may struggle to attend care of any form after rape as the attendance reminds them of the trauma.[67 68] This will affect retention overall.

We are aware of the limitation with regards to generalisability of findings to all rape survivors, as low levels of reporting of rape to the police is a well-known phenomenon globally[69]. Women who report a rape are likely to be different in several aspects from the wider group of women who experience rape. Those who report are more likely to have experienced injuries, to have had witnesses, or to be raped by non-partners (strangers). However, these are also women who are accessible from the wider population of women who are raped for longitudinal research on health outcomes. Women who come forward to the police or to health services are also the group who require services and so are an important group to study. From a pragmatic point of view, the methodology employed here is justifiable, especially in light of the logistical difficulty and high cost of recruiting a population-based cohort. Generalizability may also be impacted on by the non-random, non-census nature of recruitment. Women may decline to participate in the study and this could introduce bias. We will aim to reduce this bias but will have to be cognisant of voluntariness of consent to participate. It is also possible that some women who decline participation may do so on account of pre-existing mental health problems.

Our description of the retention and linkage cascade in HIV+ women and the impact of rape will provide very valuable new knowledge that we hope will be of value to HIV health services. We anticipate being unable to scrutinise risk factors for loss to HIV care in the current sample or to compare risk factors between the two arms. This will be an area of important future research should the study demonstrate that rape is an important barrier for optimal HIV treatment and retention in care.

CONCLUSION

Our study will enable us to build scientific knowledge to answer critical questions related to the health impact of rape. It is will be among the first to determine the extent of women's HIV risk elevation in the medium- and long- term post-rape, and physical and mental morbidity and the potential interactions between these. We will also be able to describe changes in morbidity attributable to legal processes (progression of the case through the criminal justice system) post-rape. Very little research has been done on medium and long-term post-rape morbidity, yet this information is critical for planning services, in particular mental health preventive and treatment interventions.

For peer review only

ACKNOWLEDGMENTS

We thank the South African National Department of Health, The Kwa-Zulu Natal Department of Health and the National Prosecuting Authority for assisting in accessing the participants. We also thank Life Line and Child Line for their assistance. We also thank the RICE research team for their commitment to the study and lastly we thank all the participants for their time and sharing of their lives with us.

For peer review only

COMPETING INTEREST DECLARATION

We have read and understood BMJ policy on declaration of interests and declare that we have no competing interests.

For peer review only

AUTHOR CONTRIBUTIONS

Naeemah Abrahams and Rachel Jewkes conceptualize the study and wrote the 1st draft of the proposal. Carl Lombard, Soraya Seedat, Andre Kengne, Gita Ramjee, Bronwyn Myers, Alehsha Sewnath, Shibe Mhlongo, Nasheeta Peers and Claudia Garcia-Moreno contributed to the completion of the study protocol. Naeemah Abrahams wrote the 1st draft of this manuscript and all authors contributed and approved the final version to be published

For peer review only

FUNDING

This research and the publication thereof is the result of funding provided by the South African Medical Research Council in terms of the SAMRC's Flagships Awards Project SAMRC-RFA-IFSP-01-2013/ RAPE COHORT

For peer review only

References

1. Devries KM, Mak JY, Garcia-Moreno C, et al. Global health. The global prevalence of intimate partner violence against women. Science 2013;**340**(6140):1527-8.
2. World Health Organisation. Global and regional estimates of violence against women: prevalence and health effects of intimate partner violence and non-partner sexual violence. Geneva: World Health Organisation, 2013.
3. Organisation WH. Post-Exposure Prophalaxsis to prevent HIV infection In: infection JWIGop-eptpH, ed. Geneva: WHO, 2007.
4. Jewkes R, Dunkle K, Nduna M, et al. Intimate partner violence, relationship gender power inequity, and incidence of HIV infection in young women in South Africa: a cohort study. The Lancet 2010;**367**:41-48.
5. Wood K, Maforah F, Jewkes R. 'He forced me to love him': Putting violence on adolescent sexual health agendas. Social Science and Medicine 1998;**47**(2):233-42.
6. Pettifor AE, Kleinschimdt I, Levin JB, et al. A community-based study to examine the effect of a youth HIV prevention intervention on young people aged 15-24 in South Africa: results of the baseline survey. Tropical Medicine & International Health 2005;**10**(10):971-80.
7. Dunkle K, Jewkes R, Brown HC, et al. Transactional sex among women in Soweto, South Africa: prevalence, risk factors and association with HIV infection. Social Science & Medicine 2004;**59**:1581-92.
8. Jewkes R, Dunkle K, Nduna M, et al. Transactional sex and HIV incidence in a cohort of young women in the Stepping Stones trial. Journal of AIDS & Clinical Research 2012;**3**:158.
9. Jewkes R, Sikweyiya Y, Morrell R, et al. The relationship between intimate partner violence, rape and HIV amongst South African men: A cross-sectional study. PLoS One 2011;**6**(9).
10. Decker MR, Seage GR 3rd, Hemenway D, et al. Intimate partner violence functions as both a risk marker and risk factor for women's HIV infection: findings from Indian husband-wife dyads. . J Acquir Immune Defic Syndr 2009;**51**:593-600.
11. Dunkle KL, Jewkes RK, Nduna M, et al. Perpetration of partner violence and HIV risk behaviour among young men in the rural Eastern Cape, South Africa [Article], 2006.
12. Jewkes R, Dunkle K, Koss MP, et al. Rape perpetration by young, rural South African men: Prevalence, patterns and risk factors. Soc Sci Med 2006;**63**(11):2949-61.
13. Abrahams N, R J, Mathews S. Depressive symptoms after a sexual assualt among women, understanding victim-perpetrator relationship and the role of social percpetions. African Journal of Psychaitry 2013;**16**(4):288-93.
14. Hembree EA, Street GP, Riggs DS, et al. Do assault-related variables predict response to cognitive behavioral treatment for PTSD? J Consult Clin Psychol 2004;**72**(3):531-4.
15. Jewkes R, Abrahams N. The epidemiology of rape and sexual coercion in South Africa: an overview. Social Science & Medicine 2002;**55**:153-66.
16. Ehlert U, Gaab J, Heinrichs M. Psychoneuroendocrinological contributions to the etiology of depression, posttraumatic stress disorder, and stress-related bodily disorders: the role of the hypothalamus–pituitary–adrenal axis. Biological psychology 2001;**57**(1):141-52.
17. Altemus M, Cloitre M, Dhabhar FS. Enhanced cellular immune response in women with PTSD related to childhood abuse. American Journal of Psychiatry 2003.
18. Bomyea J, Risbrough V, Lang AJ. A consideration of select pre-trauma factors as key vulnerabilities in PTSD. Clin Psychol Rev 2012;**32**(7):630-41.
19. Prathikanti S, Weinberger DR. Psychiatric genetics – the new era: genetic research and some clinical implications. British Medical Bulletin 2005;**73-74**(1):107-22.
20. Elklit A, Christiansen DM. ASD and PTSD in Rape Victims. Journal of Interpersonal Violence 2010.
21. Zinzow HM, Resnick HS, McCauley JL, et al. The role of rape tactics in risk for posttraumatic stress disorder and major depression: Results from a national sample of college women. Depress Anxiety 2010;**27**(8):708-15.

For peer review only

22. Rakyan VK, Down TA, Balding DJ, et al. Epigenome-wide association studies for common human diseases. Nature Reviews Genetics 2011;**12**(8):529-41.
23. Coker AL, Davis KE, Arias I, et al. Physical and mental health effects of intimate partner violence for men and women. Am J Prev Med 2002;**23**(4):260-68.
24. Keeshin BR, Cronholm PF, Strawn JR. Physiologic changes associated with violence and abuse exposure: an examination of related medical conditions. Trauma, Violence, & Abuse 2012;**13**(1):41-56.
25. Logan JG, Barksdale DJ. Allostasis and allostatic load: expanding the discourse on stress and cardiovascular disease. J Clin Nurs 2008;**17**(7b):201-08.
26. Golding JM. Intimate partner violence as a risk factor for mental disorders: A meta-analysis. Journal of family violence 1999;**14**(2):99-132.
27. Rosengren A, Hawken S, Ounpuu S, et al. Association of psychosocial risk factors with risk of acute myocardial infarction in 11119 cases and 13648 controls from 52 countries (the INTERHEART study): case-control study. Lancet 2004;**364**(9438):953-62.
28. Rozanski A, Blumenthal JA, Kaplan J. Impact of psychological factors on the pathogenesis of cardiovascular disease and implications for therapy. Circulation 1999;**99**(16):2192-217.
29. Das S, O'Keefe JH. Behavioral cardiology: recognizing and addressing the profound impact of psychosocial stress on cardiovascular health. Current hypertension reports 2008;**10**(5):374-81.
30. UNAIDS. *Report on the Global AIDS epidemic*. Geneva: UNAIDS, 2012.
31. Pettifor A, MacPhail C, Suchindran S, et al. Factors associated with HIV testing among public sector clinic attendees in Johannesburg, South Africa. AIDS Behav 2010;**14**(4):913-21.
32. Chi BH, Yiannoutsos CT, Westfall AO, et al. Universal definition of loss to follow-up in HIV treatment programs: a statistical analysis of 111 facilities in Africa, Asia, and Latin America. PLoS Med 2011;**8**(10):e1001111.
33. Nachega JB, Mutamba B, Basangwa D, et al. Severe mental illness at ART initiation is associated with worse retention in care among HIV-infected Ugandan adults. Tropical Medicine & International Health 2012;**Nov 8. doi: 10.1111/tmi.12019**.
34. Cornell M, Schomaker M, Garone DB, et al. Gender differences in survival among adult patients starting antiretroviral therapy in South Africa: a multicentre cohort study. PLoS Med 2012;**9**(9):e1001304.
35. Machisa M, Jewkes R, Lowe Morna C, et al. The war @ home: Preliminary findings of the Gauteng Gender Violence Prevalence Study Gauteng: Gender Links, 2011.
36. UNAIDS. Global AIDS Response: Country Progress Report 2012, Republic of South Africa. Pretoria: Republic of South Africa, 2012.
37. Wand H, Ramjee G. Assessing and evaluating the combined impact of behavioural and biological risk factors for HIV seroconversion in a cohort of South African women. AIDS Care 2012;**24**(9):1155-62.
38. Ramjee G, Coumi N, Dladla-Qwabe N, et al. Research Experiences in conducting multiple community-based HIV prevention trials among women in KwaZulu-Natal, South Africa. AIDS research and therapy 2010;**7**(10).
39. UNICEF. Thuthuzela Care Centres 2012 [Available from: http://www.unicef.org/southafrica/hiv_aids_998.html. .
40. Abrahams N, Jewkes R. 'I believe I should not take the pills as people who are HIV positive take them': Barriers to Post Exposure Prophylaxis (PEP) Completion After Rape in South Africa. Cult Health Sex 2010;**12**:471-84.
41. Abrahams N, Jewkes R, Lombard C, et al. Impact of telephonic psycho-social support on adherence to post exposure prophylaxis (PEP) after rape: a randomised controlled trial. AIDS Care 2010;**16**:1-9.

For peer review only

42. Garcia-Moreno C, Jansen HAFM, Ellsberg M, et al. WHO Multi-country Study on Women's Health and Domestic Violence against Women: Initial results on prevalence, health outcomes and women's responses. Geneva, Switzerland: World Health Organisation, 2005.
43. Jewkes R, Nduna M, Levin J, et al. A cluster randomised controlled trial to determine the effectiveness of Stepping Stones in preventing HIV infections and promoting safer sexual behaviour amongst youth in the rural Eastern Cape, South Africa: trial design, methods and baseline findings. Tropical Medicine and International Health 2006;**11**:3-16.
44. Jewkes R, Christofides N, Vetten L, et al. Medico-legal findings, legal case progression, and outcomes in South African rape cases: retrospective review. PLoSMed 2009;**6**(10):e1000164.
45. Vaux A, Phillips J, Holly L, et al. The Social Support Appraisals (SS-A) Scale: Studies of Reliability and Validity. American Journal of Community Psychiatry 1986;**14**(2):195-219.
46. Jalbrzikowski M, Sugar CA, Zinberg J, et al. Coping styles of individuals at clinical high risk for developing psychosis. Early Interv Psychiatry 2012.
47. Singh D, Chaudoir SR, Escobar MC, et al. Stigma, burden, social support, and willingness to care among caregivers of PLWHA in home-based care in South Africa. AIDS Care 2011;**23**(7):839-45.
48. Sheehan DV, Sheehan KH, Shytle RD, et al. Reliability and validity of the Mini International Neuropsychiatric Interview for Children and Adolescents (MINI-KID). J Clin Psychiatry 2010;**71**(3):313-26.
49. Nduna M, Jewkes R, Dunkle K, et al. Associations between depressive symptoms, sexual behaviour and relationship characteristics: a prospective cohort study of young women and men in the Eastern Cape, South Africa. J Int AIDS Soc 2010;**13**:44.
50. Saunders JB, Aasland O, Babor T, et al. Development of the Alcohol Use Disorders identification test (AUDIT): WHO Collaborative project on early detection of persons with harmful alcohol consumption II. . Addiction 1993;**88**:791-804.
51. Sinadinovic K, Wennberg P, Berman AH. Population screening of risky alcohol and drug use via Internet and Interactive Voice Response (IVR): a feasibility and psychometric study in a random sample. Drug and alcohol dependence 2011;**114**(1):55-60.
52. Vythilingum B, Roos A, Faure SC, et al. Risk factors for substance use in pregnant women in South Africa. S Afr Med J 2012;**102**(11):851-4.
53. Gray MJ, Litz BT, Hsu JL, et al. Psychometric properties of the life events checklist. Assessment 2004;**11**(4):330-41.
54. Davidson JR, Book SW, Colket JT, et al. Assessment of a new self-rating scale for post-traumatic stress disorder. Psychol Med 1997;**27**(1):153-60.
55. Zlotnick C, Davidson J, Shea MT, et al. Validation of the Davidson Trauma Scale in a sample of survivors of childhood sexual abuse. J Nerv Ment Dis 1996;**184**(4):255-7.
56. Bernstein D, Stein J, Newcomb M, et al. Development and validation of a brief screening version of the Childhood Trauma Questionnaire. Child Abuse & Neglect 2003;**27**:169-90.
57. Jewkes R, Dunkle K, Nduna M, et al. Associations between childhood adversity and depression, substance abuse & HIV & HSV2 in rural South African youth. Child Abuse and Neglect 2010;**34**(11):833-41.
58. Jewkes R, Levin J B, Penn-Kekana L. A. Gender inequalities, intimate partner violence and HIV preventive practices: findings of a South African cross-sectional study. Soc Sci Med 2003;**56**(1):125-34.
59. Department of Health. The 1998 South African Demographic and Health Survey. Full Report. . Pretoria: Department of Health, 1999.
60. World Health Organisation. The WHO STEPwise approach to chronic disease risk factor surveillance (STEPS) (instrument v2.1). Geneva: World Health Organization.
61. Organisation WH. Consolildated Guidelines on HIV testing services Geneva: World Health Organisation, 2015.

For peer review only

62. World Health Organization. *Putting Women First: Ethical and Safety Recommendations for Research on Domestic Violence Against Women*. Geneva, Switzerland: World Health Organization, 2001.
63. Shishana O, Rehle T, Simbayi L, et al. South African HIV Prevalence, Incidence and Behavioural survey 2012. Cape Town Human Sceinces Research Council, 2014.
64. Abrahams N, Gevers A. A rapid appraisal of the status of mental health support in post-rape care services in the Western Cape. 2017 2017;**23**(1).
65. Geng EH, Bangsberg DR, Musinguzi N, et al. Understanding Reasons for and Outcomes of Patients Lost to Follow-Up in Antiretroviral Therapy Programs in Africa Through a Sampling-Based Approach. Journal of acquired immune deficiency syndromes (1999) 2010;**53**(3):405-11.
66. Rosen S, Fox MP, Gill CJ. Patient Retention in Antiretroviral Therapy Programs in Sub-Saharan Africa: A Systematic Review. PLoS Medicine 2007;**4**(10):e298.
67. Nesvold H, Friis S, Ormstad K. Sexual assault centers: attendance rates, and differences between early and late presenting cases. Acta Obstet Gynecol Scand 2008;**87**(7):707-15.
68. Jones JS, Alexander C, Wynn BN, et al. Why women don't report sexual assault to the police: The influence of psychosocial variables and traumatic injury. The Journal of emergency medicine 2009;**36**(4):417-24.
69. Kelly L, Lovett J, Regan L. *A gap or a chasm? Attrition in reported rape cases*. London: Home Office Research, Development and Statistics Directorate, 2005.

For peer review only

A study protocol on the impact of rape on women's health, on the use of health services and on their experiences of violence: A South African longitudinal study

Journal:	*BMJ Open*
Manuscript ID	bmjopen-2017-017296.R1
Article Type:	Protocol
Date Submitted by the Author:	09-Jun-2017
Complete List of Authors:	Abrahams, Naeemah; South African Medical Research Council, Gender & Health Research Unit<br>Seedat, Soraya; Stellenbosch University, Department of Psychiatry<br>Lombard, Carl; South Africa Medical Research Council, Biostatistics Unit<br>Kengne , AP; South African Medical Research Council,<br>Myers, Bronwyn; South African Medical Research Council, Alcohol, Tobacco and Other Drug Research Unit<br>Sewnath, Alesha; South African Medical Research Council, Gender& Health Research Council<br>Mhlongo, Shibe; South African Medical Research Council, Gender & Health Research Unit<br>Ramjee, Gita; HIV Prevention Unit, Medical Research Council; HIV Prevention Unit, Medical Research Council<br>Peer, Nasheeta; South African Medical Research Council, Non-Communicable Disease Research Unit<br>Garcia-Moreno, Claudia; World Health Organisation, Department of Reproductive Health and Research<br>Jewkes, Rachel; South African Medical Research Council, Gender & Health Research Unit
**Primary Subject Heading**:	Public health
Secondary Subject Heading:	HIV/AIDS
Keywords:	Rape, MENTAL HEALTH, Gender Based Violence

Title: Study protocol for a longitudinal study evaluating the impact of rape on women's health and their use of health services in South Africa

Authors:
Naeemah Abrahams (corresponding author): Gender & Health Research Unit
South African Medical Research Council
P O Box 19070 Tygerberg
Cape Town, 7405
Tel: 021 9380823; Email: nabraham@mrc.ac.za
Fax: 021 9380310

Soraya Seedat: Anxiety and Stress Disorder Unit
University of Stellenbosch
Cape Town, South Africa
Tel: +27 21 9389161; Email: sseedat@sun.ac.za

Carl Lombard: Biostatistics Unit
South African Medical Research Council
Cape Town, South Africa
Tel: +27 21 9380328; Email: carl.lombard@mrc.ac.za

Andre Kengne: Non-Communicable Disease Research Unit
South African Medical Research Council
Cape Town, South Africa
Tel: +27 21 9380841; Email: Andre.Kengne@mrc.ac.za

Bronwyn Myers: Alcohol, Tobacco and Other Drug Research Unit
South African Medical Research Council
Cape Town, South Africa
Tel: +27 21 9380993; Email: bmyers@mrc.ac.za

Alesha Sewnath: Gender & Health Research Unit
South African Medical Research Council
Durban, South Africa
Tel: +27 31 2423721; Email: Alesha.Sewnath@mrc.ac.za

Shibe Mhlongo: Gender & Health Research Unit
South African Medical Research Council
Cape Town, South Africa
Tel: +27 31 9380826; Email: Shibe.Mhlongo@mrc.ac.za

Gita Ramjee: HIV Prevention Research Unit
South African Medical Research Council
Durban, South Africa
Tel: +27 31 2423635; Email: gita.ramjee@mrc.ac.za

Nasheeta Peer: Non-Communicable Disease Research Unit

For peer review only

For peer review only - http://bmjopen.bmj.com/site/about/guidelines.xhtml

South African Medical Research Council
Durban, South Africa
Tel: 27 31 2034882; E-mail: nasheeta.peer@mrc.ac.za

Claudia Garcia-Moreno: Department of Reproductive Health and Research, World Health Organization
Geneva, Switzerland
Tel: +41 22 791 4353; Email: garciamorenoc@who.int

Prof Rachel Jewkes: Gender & Health Research Unit
South African Medical Research Council
Pretoria, South Africa
Tel: 0123398525; Email: rjewkes@mrc.ac.za

For peer review only

For peer review only - http://bmjopen.bmj.com/site/about/guidelines.xhtml

INTRODUCTION

South Africa is a country known for its high levels of HIV infection and sexual violence. Although the interface between gender-based violence, HIV and mental health has been described, there are substantial gaps in knowledge of the medium- and long-term health impact. The 2010 Global Burden of Disease study excluded many health outcomes associated with rape and other forms of gender-based violence because systematic reviews revealed huge gaps in data and poor evidence of health effects. This study aims to describe the incidence and attributable burden of physical and mental health problems (including HIV acquisition) in adult women over a two-year post-rape period, through comparison with a cohort of women who have not been raped. The study will substantially advance our understanding of the impact of rape and will generate robust data to assist in the development of post-rape health services and the delivery of evidence-based care.

Methods and analysis

This longitudinal study seeks to recruit 1 008 rape-exposed and 1 008 rape non-exposed women. Women were recruited from health services and assessments were carried out at baseline, 3, 6, 9, 12, 18 and 24 months. Outcome measures include exposure to risk factors; mental health status; cardio-metabolic risks; and biomarkers for HIV, STIs, pregnancy and stress. The primary analysis will be to compare HIV incidence in the two groups using log-rank tests. Appropriate models to predict health outcomes over time will also be applied.

Ethics and dissemination

The South African Medical Research Council's Ethics Committee approved the study. As rape is a key element of the study, the safety and protection of participants guides the research process. We will adopt a research uptake strategy to ensure dissemination to policy makers, service providers and advocacy groups. Peer-review journal articles will be published.

Keywords: rape, HIV, health impact

For peer review only - http://bmjopen.bmj.com/site/about/guidelines.xhtml

STRENGTHS AND LIMITATIONS OF THE STUDY

- The study will build knowledge in an area with many gaps and will contribute to describing the health burden of gender-based violence.
- The study uses a longitudinal design with a large sample, and combines biological and behavioural data.
- The study is conducted in a developing setting.
- Recruiting only rape survivors who report rape means that our study findings will not be generalisable to all rape survivors.
- Sexual violence is often under-reported and may not be revealed by participants in the non-exposed group at screening and enrolment.

BACKGROUND

Lifetime exposure to rape and other forms of sexual violence is relatively high in most countries, as reported in global prevalence studies released in June 2013.[1 2]Although aspects of the health impact of rape have been described, the data available has major limitations. Systematic reviews to establish the magnitude of health effects associated with exposure to physical and sexual intimate partner violence (IPV) and non-partner sexual violence, including rape, indicate that the current evidence is overwhelmingly based on cross-sectional studies and/or clinical populations.[2] Furthermore, the conditions studied are mostly limited to mental ill-health and aspects of reproductive health. It is thus difficult to quantify the health burden in the absence of high-quality outcome data from longitudinal studies.

The prevention of post-rape HIV acquisition has been one of the main targets of health care following a rape.[3] However, the pathways to post-rape HIV acquisition and health outcomes have yet to be fully elucidated. While HIV can be acquired during a rape event, rape may indirectly elevate HIV acquisition risk in the medium and long term through other possible pathways. Evidence of these pathways stems from research on IPV and HIV.[4] One pathway is via acquiescence, where exposure to IPV reduces women's ability to assert themselves in sexual contexts, and determine the timing and circumstances of sex, including condom use and the insistence on fidelity.[5 6] The second pathway is through elevated high-risk, sexual behaviour on the women's part, including engagement in sex work, transactional sex, seeking older partners and having multiple partners.[6-8] The third pathway is that while women themselves may not change their behaviour, they may sustain or be drawn to relationships with men who are at higher risk of being HIV positive than other men. There is evidence that men who use violence towards women, including rape, are riskier, have more partners, have more sexually transmitted infections (STIs) and are more likely to be HIV positive. Similarly, men are more likely to partner more vulnerable women.[9-12]

It is likely that these pathways are important post rape and that psychological reaction to rape and rape stigma are powerful mediators of these pathways. These are, in turn, influenced by the circumstances of the rape. There is some evidence that self-blame results in greater and more enduring psychological impact, and poorer post-traumatic stress disorder (PTSD) treatment responses.[13 14] Evidence from rape counselling services suggests that court processes after rape may be particularly stressful for women,[15] but the health impact in general has not been empirically described. Research is needed to test these pathways described above in relation to rape exposure to enable a better understanding of their relative importance. This is critical for developing tailored HIV prevention interventions. Furthermore, there is a need for research that combines clinical assessments of post-rape mental health (i.e. PTSD and depression) with biological markers of stress. The prolonged release of cortisol in the body has been linked to several psychiatric disorders, including PTSD,[16] and it has been suggested that

For peer review only - http://bmjopen.bmj.com/site/about/guidelines.xhtml

this itself may impact immune system functioning and create enhanced susceptibility to HIV infection. Further research is needed to investigate links between rape, mental ill-health and dysfunctional hypothalamic-pituitary-adrenal (HPA) axis activity.[17 18]

Evolving research is contributing to the better understanding of the role of genetics in psychiatric disorders, including responses to trauma, such as sexual violence. Psychiatric genetics has the potential to inform our understanding of neuropsychiatric disorders,[19] including the development of PTSD and other post-rape mental health outcomes, and to understand why not all survivors develop mental health disorders.[20 21] Factors other than the traumatic event are likely to contribute to their development. Environmental influences, such as trauma (rape), can trigger epigenetic changes resulting in genes being silenced or expressed.[22] Epigenetic mechanisms can therefore be a mediator between genes and the environment. Research is needed to deepen our knowledge of the role of epigenetics in the development of post-rape mental health disorders.

The relationship between non-communicable diseases (such as cardio-metabolic diseases) and rape has not been described in longitudinal studies in the literature. Suggestions of a potential relationship can be gained from cross-sectional research on the associations between physical and psychological IPV, and self-reported chronic diseases including hypertension, diabetes, asthma, heart disease, cancer and arthritis.[23] It has been suggested that the stress response following rape may lead to the development of chronic diseases and its risk factors via similar causal pathways as in individuals exposed to general psychological stress that are known mediators of poor health care.[24] Direct pathways have been hypothesised via physiological mechanisms when traumatic stressors activate a cascade of physiological responses that affect the cardiovascular, metabolic, neural, endocrine and immune systems.[24-26] These include pathways via behavioural coping mechanisms such as detrimental lifestyle choices. Research is needed to understand these associations and the potential pathways using biological markers. Many studies, using different study designs and approaches, have examined the relationship between psychosocial stress and cardiovascular disease (CVD), and its risk factors.[27-29] None of these studies have described the relationship between psychosocial stress triggered by rape exposure and the development of CVD risk factors in longitudinal studies.

In sub-Saharan Africa, an area with a high population prevalence of HIV infection, it is common for women who are HIV positive to be raped. Not much is known about HIV positive women who are raped and the specific health burden associated with such exposure. In recent years, there has been a substantial increase in HIV testing across sub-Saharan Africa, especially among women attending antenatal services, and a much greater linkage to HIV treatment services.[30 31] High levels of medication adherence and retention during treatment are essential for the effectiveness of anti-retroviral therapy in preventing HIV disease progression and mortality. Consequently, research on barriers to adherence and retention in HIV treatment and care services has been increasing.[32] Psycho-social factors are one group of factors that undermine linkage and retention in care, and among rape survivors, mental illness and stigma are of particular concern because they are recognised barriers to HIV adherence and retention in care.[33 34] Understanding and developing interventions to respond appropriately to the impact of rape on women's ability to adhere to treatment and remain in care is important for optimising women's HIV treatment outcomes.

Given the state of the knowledge on the long-term health effects of gender-based violence, it is not surprising to find few academic papers on recruitment and retention of this vulnerable population. Indeed, longitudinal studies are challenging to conduct among all study populations. The gender-based violence studies that follow participants over a period, are mainly from clinical settings, in particular for

For peer review only - http://bmjopen.bmj.com/site/about/guidelines.xhtml

management of PTSD in populations such as war veterans, or from evaluations of batterers programmes; none were found among community samples in developing settings.[35-39]

To fill these gaps in the knowledge, we are undertaking research that has the overall goal of advancing the understanding of the health consequences of rape, notably HIV and mental health. This study is particularly well suited to being conducted in South Africa because it is a country with high sexual violence and HIV incidence[9 40], and is the country with the largest population of individuals living with HIV.[41]

METHODS/DESIGN

Research aims

The study has one primary aim and eight secondary aims.

Primary aim

To determine the incidence and attributable burden of HIV acquisition in adult women up to 24 months post-rape in comparison to women who have not been raped.

Secondary aims

1. To determine the incidence, attributable burden and recovery rates of physical and mental health problems that may enhance HIV risk at 3, 6, 9 ,12, 18 and 24 months.
2. To determine the individual, relational, social and criminal justice risk factors for health problems at the different time points that may be HIV risk factors and associated with the persistence of symptoms.
3. To estimate the relative importance of the different hypothesised pathways to HIV acquisition.
4. To determine the impact of rape on HIV positive survivors' ability to link to HIV care, retention of treatment and sexual risk-taking behaviour.
5. To evaluate changes in cortisol levels and other steroid hormones (cortisone, testosterone, progesterone, corticosterone and DHEA) over time, measuring their levels in hair, and to evaluate the stress-response of women before and after the traumatic event of rape.
6. To investigate genetic and epigenetic factors as participating biomarkers in the etiology and trajectory of PTSD among rape-exposed women.
7. To determine the incidence and attributable burden of CVD risk factors, markers of increased CVD risk, and hypertension and diabetes related renal dysfunction, comparing rape-exposed and non-exposed women.
8. To explore, qualitatively, the experiences of both rape-exposed and rape non-exposed women, and to focus on motivations and experiences of the research process, retention issues and the support provided to them.

The study is called the Rape Impact Cohort Evaluation (RICE) study and is referred as this throughout this paper. This comparative cohort study includes a 24-month follow-up. The sample size calculation addresses the primary aim. This is based on a log-rank test comparing the HIV incidence between rape-exposed and non-exposed groups. To achieve 90% power to detect a significant difference in HIV incidence, assuming an annual incidence of 9% and 6% among the rape-exposed and non-exposed groups respectively, and given an expected 36-month accrual and a minimum follow-up of 12 months, at a 5% significance level and 10% dropout, 193 HIV positive incident cases will be required.

A minimum HIV negative cohort of 780 participants is required in each of the exposure groups. We will recruit at least 844 HIV-negative participants in each exposure group and the last participant recruited will be followed up for 12 months. Research in KwaZulu-Natal (KZN), where this study is being

For peer review only - http://bmjopen.bmj.com/site/about/guidelines.xhtml

undertaken, has found an average annual HIV incidence of 10% among young women participating in trials.[42] We anticipate that 20% of participants will be HIV positive at baseline and, as such, a total sample size of 2 016 (1 008 per arm) will be recruited. We anticipate a 10% drop-out based on previous experiences with cohorts in KZN, where 95–97% retention was achieved in five longitudinal studies.[43] All participants will have data collected at baseline, 3, 6, 9 and 12 months follow-up. Participants recruited earlier in the study will also have data collected post 12 months, at 6-monthly intervals up to 24 months, which will give greater power to the study.

Setting

The study setting is in and around the city of Durban in the KwaZulu-Natal (KZN) province. The rape-exposed participants are recruited from four rape services that are spread across the city of Durban. Three of the rape centres are Thuthuzela Care Centres (TCC). These are one-stop dedicated sexual assault services based at public hospitals providing 24-hour integrated care to rape survivors, including access to police, counselling and medical care, which are also described in the law.[44 45] The health services provided include provision of initial medical and forensic care, and HIV prevention is a strong component of the TCC model of care. The three TTCs from which we recruit our rape-exposed group are based at regional hospitals in the north and south of the city (names omitted to ensure confidentiality). The fourth recruitment site is the Crisis Centre at a regional hospital situated in the city centre. The Crisis Centre is not an exclusive sexual assault service as the TCCs are, but provide similar care to rape survivors. Although more TCCs are located at district hospitals in the rural areas surrounding Durban, it would have placed a great financial strain on the study to support rural participants. Also, retention is a known challenge, as reported in HIV research, if study participants are from highly mobile populations, and if the study clinic is situated some distance from where people live.[46] We were lucky to set up our research study clinic (RICE clinic) on a hospital complex in one of the big southern townships, about 20 km south of Durban city centre and within easy access to public transport. We opted to recruit only from services within a 50km radius of the study clinic. However, survivors from nearby rural areas may seek services at any of the four recruitment sites and we will recruit these people if they indicate their willingness to travel to the RICE clinic. Non-rape exposed participants are recruited from public health family planning clinics in the same vicinity as the recruitment sites of the rape-exposed women. Permission to recruit at the TCCs was granted by the National Prosecuting Authority, and the Provincial Department of Health granted permission to recruit at family planning clinics.

At recruitment, we invite women to our RICE clinic where formal informed consent procedures are conducted as well as the baseline interview and assessments (see Figure 1). We explain in detail the study procedures and the follow-up clinic visits, and offer transport cost or a transport service to and from the clinic.

Inclusion and exclusion criteria

Rape-exposed participants are women aged 16–40 years who have lodged a complaint of rape within the previous 20 days at a rape centre. Children under 16 years are excluded because they constitute a vulnerable group that requires additional ethical safeguards. The upper age limit of 40 was chosen because the HIV incidence in women is much lower over the age of 40 years. The limit of 20 days was chosen to ensure that the time points for data collection are reasonably standardised across the study population, and to allow for baseline documentation of acute stress reactions (PTSD for the current event can only be assessed at the follow-up visits) and HIV status. Participants are excluded if they are very severely mentally distressed, mentally disabled or more than 14 weeks pregnant.

For peer review only - http://bmjopen.bmj.com/site/about/guidelines.xhtml

Participants attending family planning (non-rape exposed) are matched for age (+/– 1 year) with recruited raped-exposed women. The choice of family planning clinic clients provides a sexually active control population who, like the women seeking services for rape, are also engaged with health services. Women who report lifetime exposure to sexual violence, as determined by a screening questionnaire, are excluded from the non-exposure cohort. The same exclusion criteria, as for rape-exposed women, such as mentally disabled and more than 14 weeks pregnant, are also applied. We follow the model of recruitment that has been successful in previous studies at sexual assault services in South Africa. These include a series of information giving and consent processes described below, and also described in detail elsewhere in other publications.[47 48]

Women recruited in our non-exposed group who report a rape and meet the inclusion criteria, i.e. enrolled within 20 days of the rape, will be switched to the exposed group.

Assessment of exposure and outcomes measures

Both cohort groups attend the same number of follow-up visits and have the same assessments. The interview content and outcome measures are listed in Table 1. Baseline interviews are conducted face-to-face in the language that participants are comfortable with (Zulu, English). Responses to questions at follow-up interviews are interviewer administered or self-completed, depending on the literacy levels of participants and their familiarity with computers.

Table 1: Exposure and outcome measures at each data collection time point

	Variable group	Measures	Source of measures
A	IPV exposure	Ever physical, sexual, emotional<br>Past year, post-rape	WHO multi-country study instrument[49] [tested in South Africa by Jewkes et al 2006][50]
B	Rape circumstances and physical trauma / injuries	Rape circumstances: # perpetrators, relationship to perpetrator, weapon use, degree of violence, type of rape<br>Intimidation by the perpetrator or friends.	From Jewkes et al 2010,[51](*questions to be asked after criminal case have been closed)*
C	Rape stigma and social support	Rape stigma and self-blame<br>Coping mechanisms<br>Rape disclosure<br>Social support<br>Social disruption – leaving home, school, relationship	Social Support Appraisal Scale;[52] Brief COPE;[53]<br>South African Stigma Scale[54]
D	Demographic factors	Demographic factors: age, income, education, employment, relationship status, sexual orientation	From Jewkes et al 2006[50]
E	Mental health outcomes	Depression<br>Anxiety disorders<br>PTSD<br>Suicidality<br>Other anxiety disorders<br>Alcohol & drug abuse<br>Cortisol level in hair<br>Epigenetic changes<br>Telomere length	MINI for psychosis and anxiety disorders;[55] CES-D (depression);[56] AUDIT (alcohol);[57] DUDIT (drugs);[57-59] Life Events Checklist (LEC);[60] Davidson Trauma Scale (DTS for PTSD)[61 62]<br>Cortisol in hair and epigenetics analysis from blood samples
F	Other trauma	Previous trauma: child abuse, other	Childhood trauma scale

For peer review only - http://bmjopen.bmj.com/site/about/guidelines.xhtml

		interpersonal violence or traumatic life events<br>*Follow up interview: rape, interpersonal violence or traumatic life events since the last interview*	Bernstein et al[63] modified for South Africa Jewkes et al[64]<br>Life Events Checklist; Rape and forced sex measures from Jewkes et al 2006;[50]
G	Criminal justice processes and circumstances of the rape	Case progression: case dropped, arrest, bail, guilty plea, case withdrawn, trial commenced, testimony in court given, trial outcome<br>Perceived satisfaction with the system including information given about whereabouts of accused	Jewkes et al 2010;[51] satisfaction questions to be developed<br>*(These questions will be asked once criminal case have been closed.)*
H	Acquiescence to male domination of sex	Attitudes to gender relations and perceived social norms<br>Consistent condom use<br>Relationship control scale<br>Sexual frequency	From Jewkes et al 2006;[50] attitudes developed in South Africa and validated in Jewkes et al 2003[65]
I	Own sexual risk behavior	Transactional sex<br>Partner numbers ever and past year<br>Concurrency	From Jewkes et al 2006;[50] 2010[64]
J	Partner riskiness	STIs of current partner<br>Use of IPV by current partner<br>Partners' concurrency/polygamy	From Jewkes et al 2006;[50] 2010[4]
K	Biological risk enhancers or co-factors	Hormonal contraception use at baseline and on-going<br>Pregnancy loss (*since last interview*): termination, miscarriage	Questions from the Demographic and Health Survey[66]
L	Cardio-metabolic risk assessment	Screening questions for risk assessment<br>Measurements: hip and waist, BP, BMI, glucose status (glycaemia/Hba1c) and lipid panels<br>CRP ultrasensitive (cardiac), Gamma-GT, ALT(SGPT), AST(SGOT), creatinine (serum),	Questions from the WHO STEPs questionnaire[67]
M	Health-care seeking practices post-rape health, HIV testing and adherence	Completion of PEP & STI treatment<br>Emergency contraception<br>HIV testing post-rape and previously<br>HIV disclosure<br>Attendance for health assessment<br>Commencement of ART and adherence<br>Experience of HIV-related ill health<br>Satisfaction with health-care provision	Items developed and tested in pilot
O	Reproductive and child health	Contraception, fertility, pregnancies, reproductive health<br>Child health including babies born during RICE study participation	Questions developed and adapted from DHS

Data is being collected in real time using an electronic data management system (see Figure 1). Trained staff administer the mental health diagnostic assessment, the MINI DSM-IV and diagnostic interview face-to-face.[55] The research nurse conducts the clinical assessments, collects the biological specimens (Table 2), conducts HIV testing and counselling, and administers a pregnancy test and an STI test (self-administered vulvar swab for Trichomonas, which is treated if positive). We follow the WHO recommendations for HIV rapid testing by conducting a second test on all positives as well as an ELISA

For peer review only - http://bmjopen.bmj.com/site/about/guidelines.xhtml

test (Vironostika HIV1/2 Elisa).[68] Standard pre-and post-test counselling is delivered in a culturally sensitive manner. Specimens for HSV2 testing are conducted at 6-monthly intervals from participants who are HSV2 negative at baseline. CD4 count and viral load are measured 6-monthly, as is the routine in public-sector clinics, in order to not overly influence health-seeking behaviour by observation.

Table 2: Clinical assessments and biological samples at each visit

	Baseline	3 months	6 months	9 months	12 months	18 months	24 months
Biometrics	X	X	X	X	X	X	X
Clinical assessment	X	X	X	X	X	X	X
Mental health assessment	X	X	X	X	X	X	X
HIV and trauma counselling	X	X	X	X	X	X	X
Laboratory evaluations							
HIV rapid test and ELISA	X	X	X	X	X	X	X
Pregnancy test	X	X	X	X	X	X	X
Trichomonas swab	X	X	X	X	X	X	X
HSV2	X	X	X	X	X	X	X
Glucose/lipid panels CRP ultrasensitive (cardiac), Gamma-GT, ALT(SGPT), AST(SGOT), creatinine,	X			X			X
Cortisol (*hair sample*)	X	X	X	X	X		
Genetic and epigenetic tests	X	X	X	X	X	X	X
VIROLOGY (HIV POSITIVE)							
Viral load and CD4	X		X		X		X
p-antigen 24 (*sero-converted)*	X	X	X	X	X	X	X
Storage							
Plasma	X	X	X	X	X	X	X
Serum	X	X	X	X	X	X	X

For peer review only

Follow-up interviews and retention

Participants are given a return date and telephonic contact is maintained between interviews. The study team understands that rape survivors' lives are often in flux in the immediate period after rape, and this can lead to considerable mobility and a change in contact details. The study retention team (retention counsellor, data manager and research assistants responsible for home visits) are primarily responsible for problem-solving retention barriers. However, all study staff are cognisant that their interactions with participants can influence retention. A tracking system to monitor retention has been developed and is updated daily. This allows for immediate intervention (see discussion below on retention challenges).

Data management and analysis

For peer review only - http://bmjopen.bmj.com/site/about/guidelines.xhtml

We use a biometric system to identify, track and link participants, and their different data sources (i.e. online interviews, clinical data and biological samples) across visits. A unique barcoded participating identification(PID) number is generated at enrolment, and we use barcodes on all samples and documents. Data is collected and entered in real time on a dedicated website, and monitored daily by the data manager. Data is downloaded to a secure server and kept in a password-protected system. Laboratory results are merged into the main dataset using the unique PID.

The primary analysis is to compare HIV incidence in the two groups. The log-rank test will be used to compare the groups, taking into account the interval censoring of event times that will occur due to the visit schedules. The Wilcoxon-type test will be used. To quantify the risk of infection, a Cox proportional hazards model will be fitted, and the hazard ratio and 95% confidence interval estimated. Interval censoring will also be taken into account in this inference. Mixed effects regression models will be used to model the mental health status of participants over time and for the comparison of the mean profiles between exposure groups. A random effects model for participants will be used to account for the clustering effect of the repeated measures. Mixed effects models, restricted to the rape-exposed group, will be used to evaluate the association of the individual, relational, social and criminal justice factors on mental health, and similar analyses will be conducted for different groups of interest, i.e. the HIV positive group. We will also examine a cascade model depicting the different pathways with the cohort of women who are HIV positive and have been raped, and compare it to HIV positive women who have not been raped. We will handle missing data following the guidelines for clinical studies.[69] Our primary outcome is time to HIV infection. For this analysis, everyone at risk in both cohorts will be part of the analysis, and if someone leaves the study, the time will be censored. Thus, time-to-event analysis accommodates missing data in a natural way. The one assumption is that the dropout and follow-up time will be the same in both groups. For other outcomes, i.e. mental health status, the repeated measurements will include the baseline value and the follow-up values. Again, all the information up to the point of leaving the study will be included in the analysis. The estimation will be conducted using maximum likelihood as described above. This is a different approach to accommodating missing data than multiple imputation.

It is possible that the two groups are different for important variables, which may also impact on risk for sexual violence and HIV acquisition. The control of confounding variables is therefore critical in our study. We collect a wide range of socio-economic variables and known confounding variables (see Table 1). Furthermore, we minimise confounding by choosing a control group that also uses health services, and we recruit from health services in the same area.

STUDY PROGRESS AND CHALLENGES

A pilot study to assess the feasibility of recruitment and administering the assessment schedule was conducted between June and October 2014. We have made a few changes to the initial protocol; mainly the change of the recruitment window period, and the addition of recruitment sites, children and sub studies (addition of genetics, epigenetics, cardiovascular samples and the qualitative component). Recruitment and enrolment commenced in November 2014. By April 2017, we had achieved 56.9% of the recruitment target for the rape cohort (n=574) and a slighter better rate among the non-exposed cohort (64.2%; n=647). Our recruitment rate per month among the rape-exposed cohort improved from 13 per month in 2015 to 23 per month in 2016, and this was mainly due to interventions discussed below. We expect this to improve marginally and hope to have our full rape cohort enrolled by August 2018. The expected end date of the study is August 2019 when the 12-month interviews of the last participants will be conducted.

For peer review only - http://bmjopen.bmj.com/site/about/guidelines.xhtml

Recruitment and enrollment challenges

For various reasons, our recruitment was much slower than we anticipated in the planning phase. Our initial plan was to enroll participants within 5 days of the rape event, but we found this to be a huge challenge and enrollment was very slow. The window period for enrollment was extended to 10 days and later to 20 days post-rape. We cannot enroll beyond 20 days post-rape because we assess mental health outcomes, such as PTSD, that are time sensitive to the traumatic event, as well as the assessment of HIV status at baseline, which is a key measurement.

We found that a larger proportion of patients reporting rape to the TCCs are children, which is different to the age profile statistics provided by the National Prosecuting Authority (NPA) at the planning phase of the study. We used 2011–2013 NPA data to plan enrollment strategy. We also do not recruit mentally challenged rape survivors or those who are too emotional to discuss research participation.

Our initial recruitment process was dependent on the trained TCC staff's ability to identify eligible participants, introduce the study, gain consent to be contacted by us (the researchers) and finally collect accurate contact details. The project staff then contacted the potential participant, and invited them to our study clinic for formal consent procedures and the baseline interview (see Figure 1). Although TCC staff were keen to assist us, our study was not their priority. We therefore enlisted the Life Line counselling staff, who provide the initial counselling service at the rape centres and we contribute towards their salary. However, when funding (USAID funding) for counselling services at the rape centers decreased at the end of 2015, the RICE investigators decided to employ dedicated RICE recruiters because recruitment remained low and it made financial sense to employ staff to increase the enrollment rather than to enroll over a longer period. We employed two of the Life Line counselors to cover four recruitment sites. They set up the initial appointments, and arrange the collection and transportation of the participants to our research clinic. Frequently, participants become non-contactable after agreeing to attend our research clinic for formal consent procedures and this is a challenge. Participants provide incorrect contact numbers or our calls and voicemails remain unanswered. The latter often happens when final arrangements are being made or at time of transport pick-up. Such behaviour is also noted among the non-exposed group recruited at family planning clinics, and we suspect potential participants are reluctant to inform us directly that they do not want to participate in the research. They may feel socially coerced to agree, but find a way to discontinue contact with us. We know that rape survivors' lives are difficult post-rape and they change their phone numbers (phones are sometimes lost/stolen at the time of the rape), or they relocate. We also know that their mental health status interferes in their ability to regain normality.[13 70] This is an inherent challenge in our study, and staff are sensitive to rape survivors and accept that this group of women may not want to participant in our study.

Retention challenges and responses

Retaining participants in longitudinal studies is challenging and rape survivors bring additional concerns. Indeed, abused women have been identified as amongst the 'hard to reach' populations in research, similar to drug users.[38] Retention is also not often discussed in peer-review journal articles, where study findings are the focus. We searched for lessons learnt from other studies, but could not find longitudinal studies with large rape cohorts. Similarly, the HIV/AIDS sector has always grappled with retention in cohort studies.[46 71] As indicated above, we have participants who relocate to other provinces for safety reasons or as a coping strategy, or they find jobs in other towns. We are aware survivors experience a range of emotional difficulties with varying levels of distress and coping skills, and we know receiving support and care from others assist them on their road to recovery. We also know that there is poor integration of mental health within rape services, and although women can return to

For peer review only - http://bmjopen.bmj.com/site/about/guidelines.xhtml

the rape centre, they often choose not to. Our study has a full-time qualified trauma counsellor who provides counselling support at the study clinic. Many, however, do not use this facility and we may need to understand this better in follow-up studies. We find that women from both cohorts, who are counselled at our clinic, often seek assistance for their daily struggles. Women will report that their stresses are related to their difficult lives such as trying to find a job and securing income. Counselling sessions are often about assisting them to cope with these hardships. We expected more women to withdraw from the study because the research reminded them of the incident, but to date, among the 26 women who withdrew at the time of writing this paper, only two mentioned this as the reason. There may be others who have withdrawn, but because this has not yet been communicated to us, we consider them loss to follow up (LTFU).

We have activities beyond reimbursement to support retention. These include updating locator information at each visit, consent to use social media, maintaining telephonic contacts between clinic visits, individual birthday wishes via WhatsApp, offering transport from the rape centre, and a Saturday morning clinic to accommodate students and working women. We try to provide women with a pleasant experience at the clinic, and offer manicures and nail painting. We constantly explore other ways to improve their experience, and are currently exploring offering 10–15 minutes head and neck massages. At the end of 2015, our retention rate was between 50% and 75% (depending on the visits), and we intensified our retention activities through an incremental increase in reimbursement monies for each additional visit, a present (necklace/bag/earrings) for those who attend their 12- and 24-month visit and an additional snack of a small chocolate or a packet of peanuts. We employed a retention counsellor whose primary task is to track and monitor participant attendance, and to conduct problem-solving counselling of the retention barriers that are identified. We also employed a driver for home visits, initially for once a week, which then increased this to twice a week. In January 2017, we employed two drivers. One was dedicated to conduct daily home visits to those who missed a visit, and the other was employed to transport participants. However, home visits are not always successful. Sometimes, a home address is not found or the participant is not known by the household. This may be deliberate misinformation given to us as a form of avoidance. We also find cases where participants have left their rented homes and contact is completely lost. Nonetheless, many home visits result in finding participants or getting updated telephone numbers.

We developed an early warning system of missed attendance so that home visits are carried out soon after a missed appointment. These retention and tracking activities have become more complex because the number of baseline participants and follow-up visits increase. Each of our 2016 participants are expected to attend seven times (a total of 14 056 clinic visits). We started a monthly raffle where a grocery or pamper parcel worth R150.00 (US$10) can be won. Participants enter their study number into the visit box at each visit and there is a draw at the end of each month.

Transport to the clinic remains one of the key challenges. We recruit women from a wide geographic area and our study clinic within a regional hospital complex is only accessible by very unpredictable local taxi services. Participants report spending a long time travelling. Since the start of 2017, we have been offering all women transport to the clinic, and participants are collected in the morning at their homes, the rape centre or a known agreed landmark (police station/taxi rank etc.). Some women do not use the collection service because they profit slightly from the transport reimbursement and therefore prefer to use public transport. Transport coordination requires very good communication amongst staff members, and between the staff and participants. This is possible because almost all participants agree to use their personal phones to maintain contact with us and we commonly use Whatsapp – especially when arrangements are finalised after office hours.

For peer review only - http://bmjopen.bmj.com/site/about/guidelines.xhtml

Lessons learnt and limitations

We have learnt many lessons on how to set up and manage a longitudinal study with rape victims, and we continue to learn as our study progresses. We do not expect enrollment of rape-exposed participants to increase much beyond 26 per month because there are few other options left to increase recruitment from these four recruitment sites. Broadening the recruitment to other TCC sites is not financially feasible. We must accept that some women are not assertive enough to tell us that they do not want to participate in our study, and to avoid disapproval, they agree initially and thereafter evade contact. We must also be cognisant that power imbalances between researcher and participant may also be at play. More importantly, the post-rape period is very difficult for survivors and they have to negotiate many unsympathetic environments, which may include the rape services (health, police, justice and social), unsupportive family and friends, as well as a broader social environment wherein rape stigma remains a reality.[13 47]

Loss to follow-up (LTFU) is the most common cause of attrition in cohort studies[46] and the loss of contact with the participants has caused frustration among staff. Continuous support, and allowing staff to discuss their frustrations has enabled them to continue interacting sympathetically with trauma victims and to prevent vicarious trauma. The RICE study counsellor supports staff in addition to participants. We also ensure staff have access to an expert psychologist who spends a day with them every quarter to assist them in working with traumatised rape survivors. Staff have said they are encouraged when returning participants are clearly on a healing path (i.e. they are less depressed, or accept an HIV status and are starting ART), which makes the work a rewarding experience.

Difficulties in retention is well known in cohort studies and we have learnt much in this regard. In hindsight, we should have employed the retention counsellor earlier in the study and should have placed greater effort on the home visit programme. We are continuously assessing and discussing how to improve retention, and we hope the two recent initiatives (transport service and the raffle) will attract people to return. We may not be aware of all the factors influencing retention, and we hope the data from home visits and from our qualitative study will provide some insight to assist us in future longitudinal studies with sensitive participants. This study will also allow us to explore differential retention and loss to follow-up between the two groups. Mental health may also influence retention of women who are depressed and/or anxious and/or using substances, and are reluctant to attend the study clinic. The problem that women may struggle to attend care of any form after rape is well described in the literature on rape research because the attendance reminds them of the trauma.[72 73] This will affect overall retention.

We are aware of the limitation of generalising the findings to all rape survivors because low levels of reporting rape to the police is a well-known phenomenon locally and globally.[15 74] Women who report a rape are likely to be different in several aspects from the wider group of women who experience rape and who do report it. Those who do report are more likely to have experienced injuries, to have had witnesses, or to have been raped by non-partners (strangers). However, these are also women who are accessible from the wider population of women who are raped for longitudinal research on health outcomes. Women who come forward to the police or to health services are also the group who require services and so are an important group to study. From a pragmatic point of view, the methodology employed here is justifiable, especially considering logistical difficulty and the high cost of recruiting a population-based cohort. Generalisability may also be impacted on by the non-random, non-census nature of recruitment. Women may decline to participate in the study and this could introduce bias, but we expect this to happen in both groups. We will aim to reduce this bias, but will have to be cognisant that participation is by voluntary consent. It is also possible that some women

For peer review only - http://bmjopen.bmj.com/site/about/guidelines.xhtml

who decline participation may do so because of pre-existing mental health problems. However, our control group are also women who attend health services and, in this way, the two groups are similar.

ETHICS AND DISSEMINATION

Ethical approval for the study was granted by the South African Medical Research Council's Ethics Committee. Rape is a key element of our study, and the safety of participants is paramount and guides the research process. Project staff receive extensive training on the ethics, safety and protection of participants. We use lessons learnt from previous studies of participants recruited at rape centres.[47 48] We introduce the study to participants as a *Women's Health and Wellbeing Study* to protect women from being identified as rape survivors. The study follows the WHO ethical and safety guidelines on conducting research on gender-based violence and applies good clinical practice.[75] We also draw on guidelines developed for conducting intervention research on violence against women.[76] We provide women with sufficient information as required by the SAMRC's Ethics Committee on specific areas such as biometrics, children involvement (see below), genetic samples, HIV testing, hair samples and storage of blood. There is a separate consenting process for each of the above. We developed a brochure and information sheets for participants. Our previous experience highlighted the importance of providing full information, time to reflect and testing the understanding of consent before consent forms are signed. Participants are given a clear explanation of voluntariness of participation and the option of withdrawal is given at each visit.

Our study includes 16- and 17-year old girls who are recruited at one recruitment site (Crisis Centre). We did not receive approval from the NPA to recruit under-18s at the TCCs. For the children, we seek consent from both the parent/legal guardian and the child. It is important that the children we approach understand that their attendance at the rape centre to seek assistance will be revealed to the parent/legal guardian if disclosure has not yet occurred. We are cognisant that some girls may not want their parent/guardians to know about the rape event and we will not recruit girls if they are opposed to disclosure. The same applies to 16- and 17-year old children recruited at the family planning clinics. We consider 16-year olds to have sufficient maturity and mental capacity to understand the study (this is acknowledged in the Children's Act No. 38 of 2005 as amended by Act 41 of 2007), and the information sheet for them is the same as those for adult women. We only enrol a girl if both the parent/legal guardian and the child give consent to participate. We do not seek consent for HIV testing from the parent/legal guardian because according to South African law, parental consent is not required.

The research involves minimal risk to participants. However, questions may be intrusive and trigger emotions that can cause short-term psychological distress. All staff are trained to respond to any distress during the interviews and to provide containment. A trauma counsellor, employed at the start of the study, is available to provide support to participants and staff. Women who require further assistance are referred to appropriate services. The National Prosecuting Authority trained the staff on what may/may not be discussed with rape survivors to ensure the research does not compromise the legal process. To protect the project staff from having to testify in a trial and the integrity of the legal process, the questionnaire does not ask about circumstances of the rape until the rape case has been closed, or the trial has commenced. We expect to have many cases closed either by the survivors or by the investigating team by the end of the follow-up period as shown in research on rape attrition in South Africa.[77] We also do not have a concern for recall bias as such information on the outcome of the legal process is of great importance to most people.

We do not expect any major medical risks as part of the study. Participants may find the prospect of an HIV test distressing, but we anticipate that most participants would have been tested for HIV and would

For peer review only - http://bmjopen.bmj.com/site/about/guidelines.xhtml

know their HIV status from the rape care received, from antenatal care or from family planning services. In addition, there have been major national HIV testing campaigns across the country.[78] Our research nurses are trained to carry out HIV counselling and, together with the study trauma counsellor, can provide appropriate support and referral.

Project staff monitor adverse events at each visit. A serious adverse event is defined as experiencing violence by a partner due to participation in the study, or injury or hospitalisation due to the violence, or death of a study participant for any reason. The research nurse monitors and documents the occurrence of adverse events since the last study visit, at each visit. The nurse follows appropriate procedures for notification and responds to adverse events that arise. The number of events occurring in each cohort will be enumerated to determine whether there is a differential risk between study arms. The data manager will also monitor the adverse event data. Project staff are responsible for determining whether participation in the study is related to the adverse event or not, by exploring the circumstances surrounding the event. All events are reported to the SAMRC's Ethics Committee.

All staff are trained on matters of confidentiality and this is maintained in the retention activities. Contacting participants in the follow-up period poses a particular risk as identified in the Guidelines for conducting gender-based violence longitudinal studies,[76] and we only recruit participants who understand and agree on the follow-up nature of the study. At recruitment, we identify the different ways in which we can contact the participants (SMS, WhatsApp and home visits) and this is reviewed at each visit. All information is stored on secure computers or servers, and protected by a firewall and passwords. Study data is kept separate from the forms that can link data to the participants (consent forms and retention forms). Participants are compensated with R80.00 (US$5.50) at the baseline interview with an incremental addition of R20 (US$1.30) at each follow-up visit. Participants are compensated for travel costs or transport is arranged if required. Participants are offered snacks, such as a sandwich and a cool-drink, as well a choice of a small chocolate or a packet of nuts, during the visit to the research clinic.

Our study will generate an extensive set of results of interest to a wide audience. We will adopt a research uptake strategy that will involve policy makers, service providers, advocacy groups and academia, i.e. peer-review journal articles.

For peer review only

For peer review only - http://bmjopen.bmj.com/site/about/guidelines.xhtml

FIGURE LEGEND

Figure 1: RICE study flow chart of participants and samples

For peer review only

For peer review only - http://bmjopen.bmj.com/site/about/guidelines.xhtml

ACKNOWLEDGMENTS

We thank the South African National Department of Health, The KwaZulu-Natal Department of Health and the National Prosecuting Authority for assisting in accessing the participants. We also thank Life Line and Child Line for their assistance. We also thank the RICE research team for their commitment to the study and lastly, we thank all the participants for their time and sharing their lives with us.

For peer review only

COMPETING INTEREST DECLARATION

We have read and understood BMJ policy on declaration of interests and declare that we have no competing interests.

For peer review only

For peer review only - http://bmjopen.bmj.com/site/about/guidelines.xhtml

AUTHOR CONTRIBUTIONS

Naeemah Abrahams and Rachel Jewkes conceptualise the study and wrote the first draft of the proposal. Carl Lombard, Soraya Seedat, Andre Kengne, Gita Ramjee, Bronwyn Myers, Alesha Sewnath, Shibe Mhlongo, Nasheeta Peers and Claudia Garcia-Moreno contributed to the completion of the study protocol. Naeemah Abrahams wrote the first draft of this manuscript, and all authors contributed to and approved the final version to be published.

For peer review only

For peer review only - http://bmjopen.bmj.com/site/about/guidelines.xhtml

FUNDING
This research and the publication thereof is the result of funding provided by the South African Medical Research Council in terms of the SAMRC's Flagships Awards Project SAMRC-RFA-IFSP-01-2013/ RAPE COHORT.

For peer review only

For peer review only - http://bmjopen.bmj.com/site/about/guidelines.xhtml

REFERENCES

1. Devries K, Mak J, García-Moreno C, et al. The Global Prevalence of Intimate Partner Violence Against Women. Science 2013;**340**(6140):1527-28
2. World Health Organisation. Global and regional estimates of violence against women: prevalence and health effects of intimate partner violence and non-partner sexual violence. Geneva: World Health Organisation, 2013. http://www.who.int/reproductivehealth/publications/violence/9789241564625/en/
3. World Health Organisation. Post-Exposure Prophalaxsis to prevent HIV infection. Joint WHO/ILO guidelines on post-exposure prophylaxis (PEP) to prevent HIV infection: Geneva: WHO, 2007 http://apps.who.int/iris/bitstream/10665/43838/1/9789241596374_eng.pdf
4. Jewkes R, Dunkle K, Nduna M, et al. Intimate partner violence, relationship gender power inequity, and incidence of HIV infection in young women in South Africa: a cohort study. The Lancet 2010;**367**:41-48
5. Wood K, Maforah F, Jewkes R. 'He forced me to love him': Putting violence on adolescent sexual health agendas. Social Science and Medicine 1998;**47**(2):233-42
6. Pettifor AE, Kleinschimdt I, Levin JB, et al. A community-based study to examine the effect of a youth HIV prevention intervention on young people aged 15-24 in South Africa: results of the baseline survey. Tropical Medicine & International Health 2005;**10**(10):971-80
7. Dunkle K, Jewkes R, Brown HC, et al. Transactional sex among women in Soweto, South Africa: prevalence, risk factors and association with HIV infection. Social Science & Medicine 2004;**59**:1581-92
8. Jewkes R, Dunkle K, Nduna M, et al. Transactional sex and HIV incidence in a cohort of young women in the Stepping Stones trial. Journal of AIDS & Clinical Research 2012;**3**:158 doi: doi:10.4172/2155-6113.1000158[published Online First: Epub Date]|.
9. Jewkes R, Sikweyiya Y, Morrell R, et al. The relationship between intimate partner violence, rape and HIV amongst South African men: A cross-sectional study. PLoS One 2011;**6**(9)
10. Decker MR, Seage GR 3rd, Hemenway D, et al. Intimate partner violence functions as both a risk marker and risk factor for women's HIV infection: findings from Indian husband-wife dyads. J Acquir Immune Defic Syndr 2009;**51**:593-600
11. Dunkle KL, Jewkes RK, Nduna M, et al. Perpetration of partner violence and HIV risk behaviour among young men in the rural Eastern Cape, South Africa [. Aids. 2006 Oct 24;20(16):2107-14.
12. Jewkes R, Dunkle K, Koss MP, et al. Rape perpetration by young, rural South African men: Prevalence, patterns and risk factors. Soc Sci Med 2006;**63**(11):2949-61 doi: 10.1016/j.socscimed.2006.07.027[published Online First: Epub Date]|.
13. Abrahams N, R J, Mathews S. Depressive symptoms after a sexual assault among women, understanding victim-perpetrator relationship and the role of social percpetions. African Journal of Psychaitry 2013;**16**(4):288-93
14. Hembree EA, Street GP, Riggs DS, et al. Do assault-related variables predict response to cognitive behavioral treatment for PTSD? J Consult Clin Psychol 2004;**72**(3):531-4 doi: 10.1037/0022-006x.72.3.531[published Online First: Epub Date]|.
15. Jewkes R, Abrahams N. The epidemiology of rape and sexual coercion in South Africa: an overview. Social Science & Medicine 2002;**55**:153-66
16. Ehlert U, Gaab J, Heinrichs M. Psychoneuroendocrinological contributions to the etiology of depression, posttraumatic stress disorder, and stress-related bodily disorders: the role of the hypothalamus–pituitary–adrenal axis. Biological psychology 2001;**57**(1):141-52
17. Altemus M, Cloitre M, Dhabhar FS. Enhanced cellular immune response in women with PTSD related to childhood abuse. American Journal of Psychiatry 2003 Sep 1;160(9):1705-7

For peer review only - http://bmjopen.bmj.com/site/about/guidelines.xhtml

18. Bomyea J, Risbrough V, Lang AJ. A consideration of select pre-trauma factors as key vulnerabilities in PTSD. Clin Psychol Rev 2012;**32**(7):630-41
19. Prathikanti S, Weinberger DR. Psychiatric genetics – the new era: genetic research and some clinical implications. British Medical Bulletin 2005;**73-74**(1):107-22 doi: 10.1093/bmb/ldh055[published Online First: Epub Date]|.
20. Elklit A, Christiansen DM. ASD and PTSD in Rape Victims. Journal of Interpersonal Violence 2010 doi: 10.1177/0886260509354587[published Online First: Epub Date]|.
21. Zinzow HM, Resnick HS, McCauley JL, et al. The role of rape tactics in risk for posttraumatic stress disorder and major depression: Results from a national sample of college women. Depress Anxiety 2010;**27**(8):708-15
22. Rakyan VK, Down TA, Balding DJ, et al. Epigenome-wide association studies for common human diseases. Nature Reviews Genetics 2011;**12**(8):529-41
23. Coker AL, Davis KE, Arias I, et al. Physical and mental health effects of intimate partner violence for men and women. Am J Prev Med 2002;**23**(4):260-68 doi: http://dx.doi.org/10.1016/S0749-3797(02)00514-7[published Online First: Epub Date]|.
24. Keeshin BR, Cronholm PF, Strawn JR. Physiologic changes associated with violence and abuse exposure: an examination of related medical conditions. Trauma, Violence, & Abuse 2012;**13**(1):41-56
25. Logan JG, Barksdale DJ. Allostasis and allostatic load: expanding the discourse on stress and cardiovascular disease. J Clin Nurs 2008;**17**(7b):201-08
26. Golding JM. Intimate partner violence as a risk factor for mental disorders: A meta-analysis. Journal of family violence 1999;**14**(2):99-132
27. Rosengren A, Hawken S, Ounpuu S, et al. Association of psychosocial risk factors with risk of acute myocardial infarction in 11119 cases and 13648 controls from 52 countries (the INTERHEART study): case-control study. Lancet 2004;**364**(9438):953-62 doi: 10.1016/S0140-6736(04)17019-0[published Online First: Epub Date]|.
28. Rozanski A, Blumenthal JA, Kaplan J. Impact of psychological factors on the pathogenesis of cardiovascular disease and implications for therapy. Circulation 1999;**99**(16):2192-217
29. Das S, O'Keefe JH. Behavioral cardiology: recognizing and addressing the profound impact of psychosocial stress on cardiovascular health. Current hypertension reports 2008;**10**(5):374-81
30. UNAIDS. *Report on the Global AIDS epidemic*. Geneva: UNAIDS, 2012. http://files.unaids.org/en/media/unaids/contentassets/documents/epidemiology/2012/gr2012/20121120_UNAIDS_Global_Report_2012_with_annexes_en.pdf
31. Pettifor A, MacPhail C, Suchindran S, et al. Factors associated with HIV testing among public sector clinic attendees in Johannesburg, South Africa. AIDS Behav 2010;**14**(4):913-21 doi: 10.1007/s10461-008-9462-5[published Online First: Epub Date]|.
32. Chi BH, Yiannoutsos CT, Westfall AO, et al. Universal definition of loss to follow-up in HIV treatment programs: a statistical analysis of 111 facilities in Africa, Asia, and Latin America. PLoS Med 2011;**8**(10):e1001111 doi: 10.1371/journal.pmed.1001111[published Online First: Epub Date]|.
33. Nachega JB, Mutamba B, Basangwa D, et al. Severe mental illness at ART initiation is associated with worse retention in care among HIV-infected Ugandan adults. Tropical Medicine & International Health 2012;**Nov 8. doi: 10.1111/tmi.12019**
34. Cornell M, Schomaker M, Garone DB, et al. Gender differences in survival among adult patients starting antiretroviral therapy in South Africa: a multicentre cohort study. PLoS Med 2012;**9**(9):e1001304 doi: 10.1371/journal.pmed.1001304[published Online First: Epub Date]|.
35. Rothbaum BO, Foa EB, Riggs DS, et al. A prospective examination of post-traumatic stress disorder in rape victims. Journal of Traumatic Stress 1992;**5**(3):455-75 doi: 10.1007/bf00977239[published Online First: Epub Date]|.

For peer review only - http://bmjopen.bmj.com/site/about/guidelines.xhtml

36. Foa E. Cognitive behavioural treatments have long-term benefits in female rape survivors with post-traumatic stress disorder. Evid Based Ment Health 2012;**15**(4):89 doi: 10.1136/ebmental-2012-100764[published Online First: Epub Date]|.
37. Schnurr PP, Friedman MJ, Engel CC, et al. Cognitive behavioral therapy for posttraumatic stress disorder in women - A randomized controlled trial., Jama. 2007 Feb 28;297(8):820-30.
38. Gondolf EW, Deemer C. Phoning Logistics in a Longitudinal Follow-Up of Batterers and Their Partners. Journal of Interpersonal Violence 2004;**19**(7):747-65 doi: doi:10.1177/0886260504265618[published Online First: Epub Date]|.
39. Gondolf EW. Limitations of experimental evaluation of batterer programs. Trauma, Violence, & Abuse 2001;**2**(1):79-88
40. Machisa M, Jewkes R, Lowe Morna C, et al. The war @ home: Preliminary findings of the Gauteng Gender Violence Prevalence Study Gauteng: Gender Links, 2011. http://www.mrc.ac.za/gender/gbvthewar.pdf
41. UNAIDS. Global AIDS Response: Country Progress Report 2012, Republic of South Africa. Pretoria: Republic of South Africa, 2012. http://files.unaids.org/en/dataanalysis/knowyourresponse/countryprogressreports/2012countries/ce_ZA_Narrative_Report.pdf
42. Wand H, Ramjee G. Assessing and evaluating the combined impact of behavioural and biological risk factors for HIV seroconversion in a cohort of South African women. AIDS Care 2012;**24**(9):1155-62
43. Ramjee G, Coumi N, Dladla-Qwabe N, et al. Research Experiences in conducting multiple community-based HIV prevention trials among women in KwaZulu-Natal, South Africa. AIDS research and therapy 2010;**7**(10)
44. Criminal Law (Sexual Offences and Related Matters) Ammendment Act 32 of 2007. South Africa 2007. http://www.justice.gov.za/legislation/acts/2007-032.pdf
45. UNICEF. Thuthuzela Care Centres. Secondary Thuthuzela Care Centres 2012. http://www.unicef.org/southafrica/hiv_aids_998.html. .
46. Geng EH, Bangsberg DR, Musinguzi N, et al. Understanding Reasons for and Outcomes of Patients Lost to Follow-Up in Antiretroviral Therapy Programs in Africa Through a Sampling-Based Approach. Journal of acquired immune deficiency syndromes (1999) 2010;**53**(3):405-11 doi: 10.1097/QAI.0b013e3181b843f0[published Online First: Epub Date]|.
47. Abrahams N, Jewkes R. 'I believe I should not take the pills as people who are HIV positive take them': Barriers to Post Exposure Prophylaxis (PEP) Completion After Rape in South Africa. Cult Health Sex 2010;**12**:471-84
48. Abrahams N, Jewkes R, Lombard C, et al. Impact of telephonic psycho-social support on adherence to post exposure prophylaxis (PEP) after rape: a randomised controlled trial. AIDS Care 2010;**16**:1-9
49. Garcia-Moreno C, Jansen HAFM, Ellsberg M, et al. WHO Multi-country Study on Women's Health and Domestic Violence against Women: Initial results on prevalence, health outcomes and women's responses. Geneva, Switzerland: World Health Organisation, 2005. http://www.who.int/reproductivehealth/publications/violence/24159358X/en/
50. Jewkes R, Nduna M, Levin J, et al. A cluster randomised controlled trial to determine the effectiveness of Stepping Stones in preventing HIV infections and promoting safer sexual behaviour amongst youth in the rural Eastern Cape, South Africa: trial design, methods and baseline findings. Tropical Medicine and International Health 2006;**11**:3-16
51. Jewkes R, Christofides N, Vetten L, et al. Medico-legal findings, legal case progression, and outcomes in South African rape cases: retrospective review. PLoSMed 2009;**6**(10):e1000164

For peer review only - http://bmjopen.bmj.com/site/about/guidelines.xhtml

52. Vaux A, Phillips J, Holly L, et al. The Social Support Appraisals (SS-A) Scale: Studies of Reliability and Validity. American Journal of Community Psychiatry 1986;**14**(2):195-219
53. Jalbrzikowski M, Sugar CA, Zinberg J, et al. Coping styles of individuals at clinical high risk for developing psychosis. Early Interv Psychiatry 2012 doi: 10.1111/eip.12005[published Online First: Epub Date]|.
54. Singh D, Chaudoir SR, Escobar MC, et al. Stigma, burden, social support, and willingness to care among caregivers of PLWHA in home-based care in South Africa. AIDS Care 2011;**23**(7):839-45 doi: 10.1080/09540121.2010.542122[published Online First: Epub Date]|.
55. Sheehan DV, Sheehan KH, Shytle RD, et al. Reliability and validity of the Mini International Neuropsychiatric Interview for Children and Adolescents (MINI-KID). J Clin Psychiatry 2010;**71**(3):313-26 doi: 10.4088/JCP.09m05305whi[published Online First: Epub Date]|.
56. Nduna M, Jewkes R, Dunkle K, et al. Associations between depressive symptoms, sexual behaviour and relationship characteristics: a prospective cohort study of young women and men in the Eastern Cape, South Africa. J Int AIDS Soc 2010;**13**:44
57. Saunders JB, Aasland O, Babor T, et al. Development of the Alcohol Use Disorders identification test (AUDIT): WHO Collaborative project on early detection of persons with harmful alcohol consumption-II. Addiction 1993;**88**:791-804
58. Sinadinovic K, Wennberg P, Berman AH. Population screening of risky alcohol and drug use via Internet and Interactive Voice Response (IVR): a feasibility and psychometric study in a random sample. Drug and alcohol dependence 2011;**114**(1):55-60 doi: 10.1016/j.drugalcdep.2010.09.004[published Online First: Epub Date]|.
59. Vythilingum B, Roos A, Faure SC, et al. Risk factors for substance use in pregnant women in South Africa. S Afr Med J 2012;**102**(11):851-4
60. Gray MJ, Litz BT, Hsu JL, et al. Psychometric properties of the life events checklist. Assessment 2004;**11**(4):330-41 doi: 10.1177/1073191104269954[published Online First: Epub Date]|.
61. Davidson JR, Book SW, Colket JT, et al. Assessment of a new self-rating scale for post-traumatic stress disorder. Psychol Med 1997;**27**(1):153-60
62. Zlotnick C, Davidson J, Shea MT, et al. Validation of the Davidson Trauma Scale in a sample of survivors of childhood sexual abuse. J Nerv Ment Dis 1996;**184**(4):255-7
63. Bernstein D, Stein J, Newcomb M, et al. Development and validation of a brief screening version of the Childhood Trauma Questionnaire. Child Abuse & Neglect 2003;**27**:169-90
64. Jewkes R, Dunkle K, Nduna M, et al. Associations between childhood adversity and depression, substance abuse & HIV & HSV2 in rural South African youth. Child Abuse and Neglect 2010;**34**(11):833-41
65. Jewkes R, Levin J B, Penn-Kekana L. A. Gender inequalities, intimate partner violence and HIV preventive practices: findings of a South African cross-sectional study. Soc Sci Med 2003;**56**(1):125-34
66. Department of Health. The 1998 South African Demographic and Health Survey. Full Report. . Pretoria: Department of Health, 1999. https://dhsprogram.com/pubs/pdf/FR206/FR206.pdf
67. World Health Organisation. The WHO STEPwise approach to chronic disease risk factor surveillance (STEPS) (instrument v2.1). Geneva: World Health Organization. http://www.who.int/chp/steps/STEPS_Instrument_v2.1.pdf
68. World Health Organisation. Consolildated Guidelines on HIV testing services Geneva: World Health Organisation, 2015. http://www.who.int/hiv/pub/guidelines/hiv-testing-services/en/
69. Molenberghs G, Kenward M. *Missing Data in Clinical Studies*. Sussex England Wiley, 2007.
70. Abrahams N, Gevers A. A rapid appraisal of the status of mental health support in post-rape care services in the Western Cape. 2017 2017;**23**(1) doi: 10.4102/sajpsychiatry.v23i1.959[published Online First: Epub Date]|.

For peer review only - http://bmjopen.bmj.com/site/about/guidelines.xhtml

71. Rosen S, Fox MP, Gill CJ. Patient Retention in Antiretroviral Therapy Programs in Sub-Saharan Africa: A Systematic Review. PLoS Medicine 2007;**4**(10):e298 doi: 10.1371/journal.pmed.0040298[published Online First: Epub Date]|.
72. Nesvold H, Friis S, Ormstad K. Sexual assault centers: attendance rates, and differences between early and late presenting cases. Acta Obstet Gynecol Scand 2008;**87**(7):707-15
73. Jones JS, Alexander C, Wynn BN, et al. Why women don't report sexual assault to the police: The influence of psychosocial variables and traumatic injury. The Journal of emergency medicine 2009;**36**(4):417-24
74. Kelly L, Lovett J, Regan L. *A gap or a chasm? Attrition in reported rape cases*. London: Home Office Research, Development and Statistics Directorate, 2005. https://www.researchgate.net/publication/238713283
75. World Health Organization. *Putting Women First: Ethical and Safety Recommendations for Research on Domestic Violence Against Women*. Geneva, Switzerland: World Health Organization, 2001. http://www.who.int/gender/violence/womenfirtseng.pdf
76. World Health Organisation. Ethical and safety recommendations for intervention research on violence against women. Building on lessons from the WHO publication. Putting women first: ethical and safety recommendations for research on domestic violence against women. in World Health Organization, ed. Geneva, 2016. http://www.who.int/reproductivehealth/publications/violence/intervention-research-vaw/en/
77. Vetten L, Jewkes R, Fuller R, et al. Tracking Justice: The Attrition of Rape Cases Through the Criminal Justice System in Gauteng. Johannesburg: Tshwaranang Legal Advocacy Centre, 2008. https://www.csvr.org.za/docs/tracking_justice.pdf
78. Shishana O, Rehle T, Simbayi L, et al. South African HIV Prevalence, Incidence and Behavioural survey 2012. Cape Town Human Sceinces Research Council, 2014. http://www.hsrc.ac.za/uploads/pageContent/4565/SABSSM%20IV%20LEO%20final.pdf

For peer review only

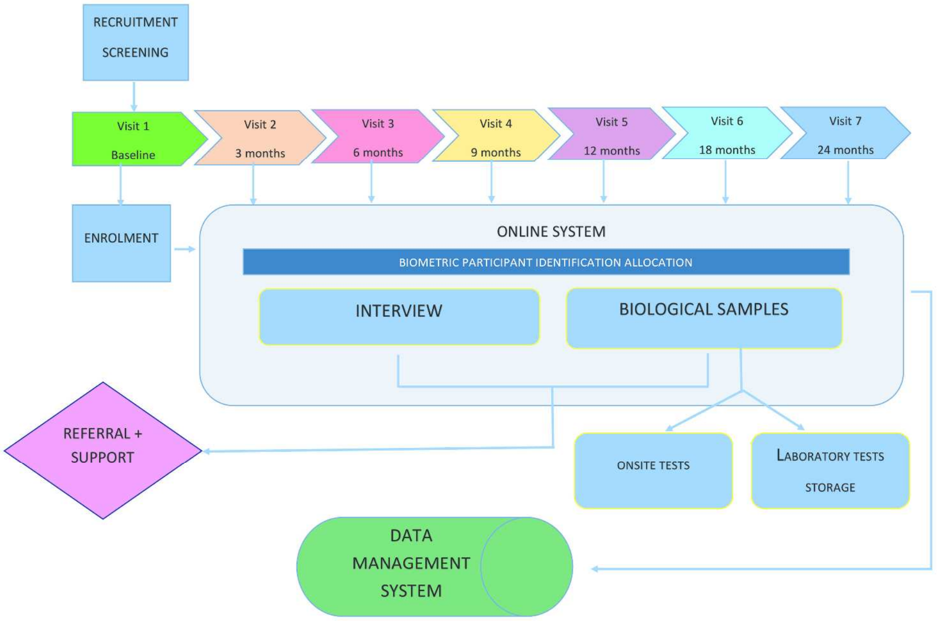

Figure 1: RICE study flow chart of participants and samples

297x209mm (300 x 300 DPI)

For peer review only - http://bmjopen.bmj.com/site/about/guidelines.xhtml

BMJ Open

Study protocol for a longitudinal study evaluating the impact of rape on women's health and their use of health services in South Africa

Journal:	*BMJ Open*
Manuscript ID	bmjopen-2017-017296.R2
Article Type:	Protocol
Date Submitted by the Author:	24-Jul-2017
Complete List of Authors:	Abrahams, Naeemah; South African Medical Research Council, Gender & Health Research Unit<br>Seedat, Soraya; Stellenbosch University, Department of Psychiatry<br>Lombard, Carl; South Africa Medical Research Council, Biostatistics Unit<br>Kengne , AP; South African Medical Research Council,<br>Myers, Bronwyn; South African Medical Research Council, Alcohol, Tobacco and Other Drug Research Unit<br>Sewnath, Alesha; South African Medical Research Council, Gender& Health Research Council<br>Mhlongo, Shibe; South African Medical Research Council, Gender & Health Research Unit<br>Ramjee, Gita; HIV Prevention Unit, Medical Research Council; HIV Prevention Unit, Medical Research Council<br>Peer, Nasheeta; South African Medical Research Council, Non-Communicable Disease Research Unit<br>Garcia-Moreno, Claudia; World Health Organisation, Department of Reproductive Health and Research<br>Jewkes, Rachel; South African Medical Research Council, Gender & Health Research Unit
**Primary Subject Heading**:	Public health
Secondary Subject Heading:	HIV/AIDS
Keywords:	Rape, MENTAL HEALTH, Gender Based Violence

SCHOLARONE™
Manuscripts

Title: Study protocol for a longitudinal study evaluating the impact of rape on women's health and their use of health services in South Africa

Authors:
Naeemah Abrahams (corresponding author): Gender & Health Research Unit
South African Medical Research Council
P O Box 19070 Tygerberg
Cape Town, 7405
Tel: 021 9380823; Email: nabraham@mrc.ac.za
Fax: 021 9380310

Soraya Seedat: Anxiety and Stress Disorder Unit
University of Stellenbosch
Cape Town, South Africa
Tel: +27 21 9389161; Email: sseedat@sun.ac.za

Carl Lombard: Biostatistics Unit
South African Medical Research Council
Cape Town, South Africa
Tel: +27 21 9380328; Email: carl.lombard@mrc.ac.za

Andre Kengne: Non-Communicable Disease Research Unit
South African Medical Research Council
Cape Town, South Africa
Tel: +27 21 9380841; Email: Andre.Kengne@mrc.ac.za

Bronwyn Myers: Alcohol, Tobacco and Other Drug Research Unit
South African Medical Research Council
Cape Town, South Africa
Tel: +27 21 9380993; Email: bmyers@mrc.ac.za

Alesha Sewnath: Gender & Health Research Unit
South African Medical Research Council
Durban, South Africa
Tel: +27 31 2423721; Email: Alesha.Sewnath@mrc.ac.za

Shibe Mhlongo: Gender & Health Research Unit
South African Medical Research Council
Cape Town, South Africa
Tel: +27 31 9380826; Email: Shibe.Mhlongo@mrc.ac.za

Gita Ramjee: HIV Prevention Research Unit
South African Medical Research Council
Durban, South Africa
Tel: +27 31 2423635; Email: gita.ramjee@mrc.ac.za

Nasheeta Peer: Non-Communicable Disease Research Unit

For peer review only

For peer review only - http://bmjopen.bmj.com/site/about/guidelines.xhtml

South African Medical Research Council
Durban, South Africa
Tel: 27 31 2034882; E-mail: nasheeta.peer@mrc.ac.za

Claudia Garcia-Moreno: Department of Reproductive Health and Research, World Health Organization
Geneva, Switzerland
Tel: +41 22 791 4353; Email: garciamorenoc@who.int

Prof Rachel Jewkes: Gender & Health Research Unit
South African Medical Research Council
Pretoria, South Africa
Tel: 0123398525; Email: rjewkes@mrc.ac.za

For peer review only

For peer review only - http://bmjopen.bmj.com/site/about/guidelines.xhtml

INTRODUCTION

South Africa is a country known for its high levels of HIV infection and sexual violence. Although the interface between gender-based violence, HIV and mental health has been described, there are substantial gaps in knowledge of the medium- and long-term health impact. The 2010 Global Burden of Disease study excluded many health outcomes associated with rape and other forms of gender-based violence because systematic reviews revealed huge gaps in data and poor evidence of health effects. This study aims to describe the incidence and attributable burden of physical and mental health problems (including HIV acquisition) in adult women over a two-year post-rape period, through comparison with a cohort of women who have not been raped. The study will substantially advance our understanding of the impact of rape and will generate robust data to assist in the development of post-rape health services and the delivery of evidence-based care.

Methods and analysis

This longitudinal study seeks to recruit 1 008 rape-exposed and 1 008 rape non-exposed women. Women were recruited from health services and assessments were carried out at baseline, 3, 6, 9, 12, 18 and 24 months. Outcome measures include exposure to risk factors; mental health status; cardio-metabolic risks; and biomarkers for HIV, STIs, pregnancy and stress. The primary analysis will be to compare HIV incidence in the two groups using log-rank tests. Appropriate models to predict health outcomes over time will also be applied.

Ethics and dissemination

The South African Medical Research Council's Ethics Committee approved the study. As rape is a key element of the study, the safety and protection of participants guides the research process. We will adopt a research uptake strategy to ensure dissemination to policy makers, service providers and advocacy groups. Peer-review journal articles will be published.

Keywords: rape, HIV, health impact

For peer review only - http://bmjopen.bmj.com/site/about/guidelines.xhtml

STRENGTHS AND LIMITATIONS OF THE STUDY

- The study will build knowledge in an area with many gaps and will contribute to describing the health burden of gender-based violence.
- The study uses a longitudinal design with a large sample, and combines biological and behavioural data.
- The study is conducted in a developing setting.
- Recruiting only rape survivors who report rape means that our study findings will not be generalisable to all rape survivors.
- Sexual violence is often under-reported and may not be revealed by participants in the non-exposed group at screening and enrolment.

BACKGROUND

Lifetime exposure to rape and other forms of sexual violence is relatively high in most countries, as reported in global prevalence studies released in June 2013.[1 2]Although aspects of the health impact of rape have been described, the data available has major limitations. Systematic reviews to establish the magnitude of health effects associated with exposure to physical and sexual intimate partner violence (IPV) and non-partner sexual violence, including rape, indicate that the current evidence is overwhelmingly based on cross-sectional studies and/or clinical populations.[2] Furthermore, the conditions studied are mostly limited to mental ill-health and aspects of reproductive health. It is thus difficult to quantify the health burden in the absence of high-quality outcome data from longitudinal studies.

The prevention of post-rape HIV acquisition has been one of the main targets of health care following a rape.[3] However, the pathways to post-rape HIV acquisition and health outcomes have yet to be fully elucidated. While HIV can be acquired during a rape event, rape may indirectly elevate HIV acquisition risk in the medium and long term through other possible pathways. Evidence of these pathways stems from research on IPV and HIV.[4] One pathway is via acquiescence, where exposure to IPV reduces women's ability to assert themselves in sexual contexts, and determine the timing and circumstances of sex, including condom use and the insistence on fidelity.[5 6] The second pathway is through elevated high-risk, sexual behaviour on the women's part, including engagement in sex work, transactional sex, seeking older partners and having multiple partners.[6-8] The third pathway is that while women themselves may not change their behaviour, they may sustain or be drawn to relationships with men who are at higher risk of being HIV positive than other men. There is evidence that men who use violence towards women, including rape, are riskier, have more partners, have more sexually transmitted infections (STIs) and are more likely to be HIV positive. Similarly, men are more likely to partner more vulnerable women.[9-12]

It is likely that these pathways are important post rape and that psychological reaction to rape and rape stigma are powerful mediators of these pathways. These are, in turn, influenced by the circumstances of the rape. There is some evidence that self-blame results in greater and more enduring psychological impact, and poorer post-traumatic stress disorder (PTSD) treatment responses.[13 14] Evidence from rape counselling services suggests that court processes after rape may be particularly stressful for women,[15] but the health impact in general has not been empirically described. Research is needed to test these pathways described above in relation to rape exposure to enable a better understanding of their relative importance. This is critical for developing tailored HIV prevention interventions. Furthermore, there is a need for research that combines clinical assessments of post-rape mental health (i.e. PTSD and depression) with biological markers of stress. The prolonged release of cortisol in the body has been linked to several psychiatric disorders, including PTSD,[16] and it has been suggested that

For peer review only - http://bmjopen.bmj.com/site/about/guidelines.xhtml

this itself may impact immune system functioning and create enhanced susceptibility to HIV infection. Further research is needed to investigate links between rape, mental ill-health and dysfunctional hypothalamic-pituitary-adrenal (HPA) axis activity.[17 18]

Evolving research is contributing to the better understanding of the role of genetics in psychiatric disorders, including responses to trauma, such as sexual violence. Psychiatric genetics has the potential to inform our understanding of neuropsychiatric disorders,[19] including the development of PTSD and other post-rape mental health outcomes, and to understand why not all survivors develop mental health disorders.[20 21] Factors other than the traumatic event are likely to contribute to their development. Environmental influences, such as trauma (rape), can trigger epigenetic changes resulting in genes being silenced or expressed.[22] Epigenetic mechanisms can therefore be a mediator between genes and the environment. Research is needed to deepen our knowledge of the role of epigenetics in the development of post-rape mental health disorders.

The relationship between non-communicable diseases (such as cardio-metabolic diseases) and rape has not been described in longitudinal studies in the literature. Suggestions of a potential relationship can be gained from cross-sectional research on the associations between physical and psychological IPV, and self-reported chronic diseases including hypertension, diabetes, asthma, heart disease, cancer and arthritis.[23] It has been suggested that the stress response following rape may lead to the development of chronic diseases and its risk factors via similar causal pathways as in individuals exposed to general psychological stress that are known mediators of poor health care.[24] Direct pathways have been hypothesised via physiological mechanisms when traumatic stressors activate a cascade of physiological responses that affect the cardiovascular, metabolic, neural, endocrine and immune systems.[24-26] These include pathways via behavioural coping mechanisms such as detrimental lifestyle choices. Research is needed to understand these associations and the potential pathways using biological markers. Many studies, using different study designs and approaches, have examined the relationship between psychosocial stress and cardiovascular disease (CVD), and its risk factors.[27-29] None of these studies have described the relationship between psychosocial stress triggered by rape exposure and the development of CVD risk factors in longitudinal studies.

In sub-Saharan Africa, an area with a high population prevalence of HIV infection, it is common for women who are HIV positive to be raped. Not much is known about HIV positive women who are raped and the specific health burden associated with such exposure. In recent years, there has been a substantial increase in HIV testing across sub-Saharan Africa, especially among women attending antenatal services, and a much greater linkage to HIV treatment services.[30 31] High levels of medication adherence and retention during treatment are essential for the effectiveness of anti-retroviral therapy in preventing HIV disease progression and mortality. Consequently, research on barriers to adherence and retention in HIV treatment and care services has been increasing.[32] Psycho-social factors are one group of factors that undermine linkage and retention in care, and among rape survivors, mental illness and stigma are of particular concern because they are recognised barriers to HIV adherence and retention in care.[33 34] Understanding and developing interventions to respond appropriately to the impact of rape on women's ability to adhere to treatment and remain in care is important for optimising women's HIV treatment outcomes.

Given the state of the knowledge on the long-term health effects of gender-based violence, it is not surprising to find few academic papers on recruitment and retention of this vulnerable population. Indeed, longitudinal studies are challenging to conduct among all study populations. The gender-based violence studies that follow participants over a period, are mainly from clinical settings, in particular for

For peer review only - http://bmjopen.bmj.com/site/about/guidelines.xhtml

management of PTSD in populations such as war veterans, or from evaluations of batterers programmes; none were found among community samples in developing settings.[35-39]

To fill these gaps in the knowledge, we are undertaking research that has the overall goal of advancing the understanding of the health consequences of rape, notably HIV and mental health. This study is particularly well suited to being conducted in South Africa because it is a country with high sexual violence and HIV incidence[9 40], and is the country with the largest population of individuals living with HIV.[41]

METHODS/DESIGN

Research aims

The study has one primary aim and eight secondary aims.

Primary aim

To determine the incidence and attributable burden of HIV acquisition in adult women up to 24 months post-rape in comparison to women who have not been raped.

Secondary aims

1. To determine the incidence, attributable burden and recovery rates of physical and mental health problems that may enhance HIV risk at 3, 6, 9 ,12, 18 and 24 months.
2. To determine the individual, relational, social and criminal justice risk factors for health problems at the different time points that may be HIV risk factors and associated with the persistence of symptoms.
3. To estimate the relative importance of the different hypothesised pathways to HIV acquisition.
4. To determine the impact of rape on HIV positive survivors' ability to link to HIV care, retention of treatment and sexual risk-taking behaviour.
5. To evaluate changes in cortisol levels and other steroid hormones (cortisone, testosterone, progesterone, corticosterone and DHEA) over time, measuring their levels in hair, and to evaluate the stress-response of women before and after the traumatic event of rape.
6. To investigate genetic and epigenetic factors as participating biomarkers in the etiology and trajectory of PTSD among rape-exposed women.
7. To determine the incidence and attributable burden of CVD risk factors, markers of increased CVD risk, and hypertension and diabetes related renal dysfunction, comparing rape-exposed and non-exposed women.
8. To explore, qualitatively, the experiences of both rape-exposed and rape non-exposed women, and to focus on motivations and experiences of the research process, retention issues and the support provided to them.

The study is called the Rape Impact Cohort Evaluation (RICE) study and is referred as this throughout this paper. This comparative cohort study includes a 24-month follow-up. The sample size calculation addresses the primary aim. This is based on a log-rank test comparing the HIV incidence between rape-exposed and non-exposed groups. To achieve 90% power to detect a significant difference in HIV incidence, assuming an annual incidence of 9% and 6% among the rape-exposed and non-exposed groups respectively, and given an expected 36-month accrual and a minimum follow-up of 12 months, at a 5% significance level and 10% dropout, 193 HIV positive incident cases will be required.

A minimum HIV negative cohort of 780 participants is required in each of the exposure groups. We will recruit at least 844 HIV-negative participants in each exposure group and the last participant recruited will be followed up for 12 months. Research in KwaZulu-Natal (KZN), where this study is being

For peer review only - http://bmjopen.bmj.com/site/about/guidelines.xhtml

undertaken, has found an average annual HIV incidence of 10% among young women participating in trials.[42] We anticipate that 20% of participants will be HIV positive at baseline and, as such, a total sample size of 2 016 (1 008 per arm) will be recruited. We anticipate a 10% drop-out based on previous experiences with cohorts in KZN, where 95–97% retention was achieved in five longitudinal studies.[43] All participants will have data collected at baseline, 3, 6, 9 and 12 months follow-up. Participants recruited earlier in the study will also have data collected post 12 months, at 6-monthly intervals up to 24 months, which will give greater power to the study.

Setting

The study setting is in and around the city of Durban in the KwaZulu-Natal (KZN) province. The rape-exposed participants are recruited from four rape services that are spread across the city of Durban. Three of the rape centres are Thuthuzela Care Centres (TCC). These are one-stop dedicated sexual assault services based at public hospitals providing 24-hour integrated care to rape survivors, including access to police, counselling and medical care.[45] The health services provided include provision of initial medical and forensic care, and HIV prevention is a strong component of the TCC model of care. The three TTCs from which we recruit our rape-exposed group are based at regional hospitals in the north and south of the city (names omitted to ensure confidentiality). The fourth recruitment site is the Crisis Centre at a regional hospital situated in the city centre. The Crisis Centre is not an exclusive sexual assault service as the TCCs are, but provide similar care to rape survivors. Although more TCCs are located at district hospitals in the rural areas surrounding Durban, it would have placed a great financial strain on the study to support rural participants. Also, retention is a known challenge, as reported in HIV research, if study participants are from highly mobile populations, and if the study clinic is situated some distance from where people live.[46] We were lucky to set up our research study clinic (RICE clinic) on a hospital complex in one of the big southern townships, about 20 km south of Durban city centre and within easy access to public transport. We opted to recruit only from services within a 50km radius of the study clinic. However, survivors from nearby rural areas may seek services at any of the four recruitment sites and we will recruit these people if they indicate their willingness to travel to the RICE clinic. Non-rape exposed participants are recruited from public health family planning clinics in the same vicinity as the recruitment sites of the rape-exposed women. Permission to recruit at the TCCs was granted by the National Prosecuting Authority, and the Provincial Department of Health granted permission to recruit at family planning clinics.

At recruitment, we invite women to our RICE clinic where formal informed consent procedures are conducted as well as the baseline interview and assessments (see Figure 1). We explain in detail the study procedures and the follow-up clinic visits, and offer transport cost or a transport service to and from the clinic.

Inclusion and exclusion criteria

Rape-exposed participants are women aged 16–40 years who have lodged a complaint of rape within the previous 20 days at a rape centre. Children under 16 years are excluded because they constitute a vulnerable group that requires additional ethical safeguards. The upper age limit of 40 was chosen because the HIV incidence in women is much lower over the age of 40 years. The limit of 20 days was chosen to ensure that the time points for data collection are reasonably standardised across the study population, and to allow for baseline documentation of acute stress reactions (PTSD for the current event can only be assessed at the follow-up visits) and HIV status. Participants are excluded if they are very severely mentally distressed, mentally disabled or more than 14 weeks pregnant.

For peer review only - http://bmjopen.bmj.com/site/about/guidelines.xhtml

Participants attending family planning (non-rape exposed) are matched for age (+/– 1 year) with recruited raped-exposed women. The choice of family planning clinic clients provides a sexually active control population who, like the women seeking services for rape, are also engaged with health services. Women who report lifetime exposure to sexual violence, as determined by a screening questionnaire, are excluded from the non-exposure cohort. The same exclusion criteria, as for rape-exposed women, such as mentally disabled and more than 14 weeks pregnant, are also applied. We follow the model of recruitment that has been successful in previous studies at sexual assault services in South Africa. These include a series of information giving and consent processes described below, and also described in detail elsewhere in other publications.[47 48]

Women recruited in our non-exposed group who report a rape and meet the inclusion criteria, i.e. enrolled within 20 days of the rape, will be switched to the exposed group.

Assessment of exposure and outcomes measures

Both cohort groups attend the same number of follow-up visits and have the same assessments. The interview content and outcome measures are listed in Table 1. Baseline interviews are conducted face-to-face in the language that participants are comfortable with (Zulu, English). Responses to questions at follow-up interviews are interviewer administered or self-completed, depending on the literacy levels of participants and their familiarity with computers.

Table 1: Exposure and outcome measures at each data collection time point

	Variable group	Measures	Source of measures
A	IPV exposure	Ever physical, sexual, emotional<br>Past year, post-rape	WHO multi-country study instrument[49] [tested in South Africa by Jewkes et al 2006][50]
B	Rape circumstances and physical trauma / injuries	Rape circumstances: # perpetrators, relationship to perpetrator, weapon use, degree of violence, type of rape<br>Intimidation by the perpetrator or friends.	From Jewkes et al 2010,[51](*questions to be asked after criminal case have been closed)*
C	Rape stigma and social support	Rape stigma and self-blame<br>Coping mechanisms<br>Rape disclosure<br>Social support<br>Social disruption – leaving home, school, relationship	Social Support Appraisal Scale;[52] Brief COPE;[53] South African Stigma Scale[54]
D	Demographic factors	Demographic factors: age, income, education, employment, relationship status, sexual orientation	From Jewkes et al 2006[50]
E	Mental health outcomes	Depression<br>Anxiety disorders<br>PTSD<br>Suicidality<br>Other anxiety disorders<br>Alcohol & drug abuse<br>Cortisol level in hair<br>Epigenetic changes<br>Telomere length	MINI for psychosis and anxiety disorders;[55] CES-D (depression);[56] AUDIT (alcohol);[57] DUDIT (drugs);[57-59] Life Events Checklist (LEC);[60] Davidson Trauma Scale (DTS for PTSD)[61 62]<br>Cortisol in hair and epigenetics analysis from blood samples
F	Other trauma	Previous trauma: child abuse, other	Childhood trauma scale

For peer review only - http://bmjopen.bmj.com/site/about/guidelines.xhtml

		interpersonal violence or traumatic life events<br>*Follow up interview: rape, interpersonal violence or traumatic life events since the last interview*	Bernstein et al[63] modified for South Africa Jewkes et al[64]<br>Life Events Checklist; Rape and forced sex measures from Jewkes et al 2006;[50]
G	Criminal justice processes and circumstances of the rape	Case progression: case dropped, arrest, bail, guilty plea, case withdrawn, trial commenced, testimony in court given, trial outcome<br>Perceived satisfaction with the system including information given about whereabouts of accused	Jewkes et al 2010;[51]<br>satisfaction questions to be developed<br>*(These questions will be asked once criminal case have been closed.)*
H	Acquiescence to male domination of sex	Attitudes to gender relations and perceived social norms<br>Consistent condom use<br>Relationship control scale<br>Sexual frequency	From Jewkes et al 2006;[50] attitudes developed in South Africa and validated in Jewkes et al 2003[65]
I	Own sexual risk behavior	Transactional sex<br>Partner numbers ever and past year<br>Concurrency	From Jewkes et al 2006;[50] 2010[64]
J	Partner riskiness	STIs of current partner<br>Use of IPV by current partner<br>Partners' concurrency/polygamy	From Jewkes et al 2006;[50] 2010[4]
K	Biological risk enhancers or co-factors	Hormonal contraception use at baseline and on-going<br>Pregnancy loss (*since last interview*): termination, miscarriage	Questions from the Demographic and Health Survey[66]
L	Cardio-metabolic risk assessment	Screening questions for risk assessment<br>Measurements: hip and waist, BP, BMI, glucose status (glycaemia/Hba1c) and lipid panels<br>CRP ultrasensitive (cardiac), Gamma-GT, ALT(SGPT), AST(SGOT), creatinine (serum),	Questions from the WHO STEPs questionnaire[67]
M	Health-care seeking practices post-rape health, HIV testing and adherence	Completion of PEP & STI treatment<br>Emergency contraception<br>HIV testing post-rape and previously<br>HIV disclosure<br>Attendance for health assessment<br>Commencement of ART and adherence<br>Experience of HIV-related ill health<br>Satisfaction with health-care provision	Items developed and tested in pilot
O	Reproductive and child health	Contraception, fertility, pregnancies, reproductive health<br>Child health including babies born during RICE study participation	Questions developed and adapted from DHS

Data is being collected in real time using an electronic data management system (see Figure 1). Trained staff administer the mental health diagnostic assessment, the MINI DSM-IV and diagnostic interview face-to-face.[55] The research nurse conducts the clinical assessments, collects the biological specimens (Table 2), conducts HIV testing and counselling, and administers a pregnancy test and an STI test (self-administered vulvar swab for Trichomonas, which is treated if positive). We follow the WHO recommendations for HIV rapid testing by conducting a second test on all positives as well as an ELISA

For peer review only - http://bmjopen.bmj.com/site/about/guidelines.xhtml

test (Vironostika HIV1/2 Elisa).[68] Standard pre-and post-test counselling is delivered in a culturally sensitive manner. Specimens for HSV2 testing are conducted at 6-monthly intervals from participants who are HSV2 negative at baseline. CD4 count and viral load are measured 6-monthly, as is the routine in public-sector clinics, in order to not overly influence health-seeking behaviour by observation.

Table 2: Clinical assessments and biological samples at each visit

	Baseline	3 months	6 months	9 months	12 months	18 months	24 months
Biometrics	X	X	X	X	X	X	X
Clinical assessment	X	X	X	X	X	X	X
Mental health assessment	X	X	X	X	X	X	X
HIV and trauma counselling	X	X	X	X	X	X	X
Laboratory evaluations							
HIV rapid test and ELISA	X	X	X	X	X	X	X
Pregnancy test	X	X	X	X	X	X	X
Trichomonas swab	X	X	X	X	X	X	X
HSV2	X	X	X	X	X	X	X
Glucose/lipid panels CRP ultrasensitive (cardiac), Gamma-GT, ALT(SGPT), AST(SGOT), creatinine,	X			X			X
Cortisol (*hair sample*)	X	X	X	X	X		
Genetic and epigenetic tests	X	X	X	X	X	X	X
VIROLOGY (HIV POSITIVE)							
Viral load and CD4	X		X		X		X
p-antigen 24 (*sero-converted)*	X	X	X	X	X	X	X
Storage							
Plasma	X	X	X	X	X	X	X
Serum	X	X	X	X	X	X	X

Follow-up interviews and retention

Participants are given a return date and telephonic contact is maintained between interviews. The study team understands that rape survivors' lives are often in flux in the immediate period after rape, and this can lead to considerable mobility and a change in contact details. The study retention team (retention counsellor, data manager and research assistants responsible for home visits) are primarily responsible for problem-solving retention barriers. However, all study staff are cognisant that their interactions with participants can influence retention. A tracking system to monitor retention has been developed and is updated daily. This allows for immediate intervention (see discussion below on retention challenges).

Data management and analysis

For peer review only - http://bmjopen.bmj.com/site/about/guidelines.xhtml

We use a biometric system to identify, track and link participants, and their different data sources (i.e. online interviews, clinical data and biological samples) across visits. A unique barcoded participating identification(PID) number is generated at enrolment, and we use barcodes on all samples and documents. Data is collected and entered in real time on a dedicated website, and monitored daily by the data manager. Data is downloaded to a secure server and kept in a password-protected system. Laboratory results are merged into the main dataset using the unique PID.

The primary analysis is to compare HIV incidence in the two groups. The log-rank test will be used to compare the groups, taking into account the interval censoring of event times that will occur due to the visit schedules. The Wilcoxon-type test will be used. To quantify the risk of infection, a Cox proportional hazards model will be fitted, and the hazard ratio and 95% confidence interval estimated. Interval censoring will also be taken into account in this inference. Mixed effects regression models will be used to model the mental health status of participants over time and for the comparison of the mean profiles between exposure groups. A random effects model for participants will be used to account for the clustering effect of the repeated measures. Mixed effects models, restricted to the rape-exposed group, will be used to evaluate the association of the individual, relational, social and criminal justice factors on mental health, and similar analyses will be conducted for different groups of interest, i.e. the HIV positive group. We will also examine a cascade model depicting the different pathways with the cohort of women who are HIV positive and have been raped, and compare it to HIV positive women who have not been raped. We will handle missing data following the guidelines for clinical studies.[69] Our primary outcome is time to HIV infection. For this analysis, everyone at risk in both cohorts will be part of the analysis, and if someone leaves the study, the time will be censored. Thus, time-to-event analysis accommodates missing data in a natural way. The one assumption is that the dropout and follow-up time will be the same in both groups. For other outcomes, i.e. mental health status, the repeated measurements will include the baseline value and the follow-up values. Again, all the information up to the point of leaving the study will be included in the analysis. The estimation will be conducted using maximum likelihood as described above. This is a different approach to accommodating missing data than multiple imputation.

It is possible that the two groups are different for important variables, which may also impact on risk for sexual violence and HIV acquisition. The control of confounding variables is therefore critical in our study. We collect a wide range of socio-economic variables and known confounding variables (see Table 1). Furthermore, we minimise confounding by choosing a control group that also uses health services, and we recruit from health services in the same area.

STUDY PROGRESS AND CHALLENGES

A pilot study to assess the feasibility of recruitment and administering the assessment schedule was conducted between June and October 2014. We have made a few changes to the initial protocol; mainly the change of the recruitment window period, and the addition of recruitment sites, children and sub studies (addition of genetics, epigenetics, cardiovascular samples and the qualitative component). Recruitment and enrolment commenced in November 2014. By April 2017, we had achieved 56.9% of the recruitment target for the rape cohort (n=574) and a slighter better rate among the non-exposed cohort (64.2%; n=647). Our recruitment rate per month among the rape-exposed cohort improved from 13 per month in 2015 to 23 per month in 2016, and this was mainly due to interventions discussed below. We expect this to improve marginally and hope to have our full rape cohort enrolled by August 2018. The expected end date of the study is August 2019 when the 12-month interviews of the last participants will be conducted.

For peer review only - http://bmjopen.bmj.com/site/about/guidelines.xhtml

Recruitment and enrollment challenges

For various reasons, our recruitment was much slower than we anticipated in the planning phase. Our initial plan was to enroll participants within 5 days of the rape event, but we found this to be a huge challenge and enrollment was very slow. The window period for enrollment was extended to 10 days and later to 20 days post-rape. We cannot enroll beyond 20 days post-rape because we assess mental health outcomes, such as PTSD, that are time sensitive to the traumatic event, as well as the assessment of HIV status at baseline, which is a key measurement.

We found that a larger proportion of patients reporting rape to the TCCs are children, which is different to the age profile statistics provided by the National Prosecuting Authority (NPA) at the planning phase of the study. We used 2011–2013 NPA data to plan enrollment strategy. We also do not recruit mentally challenged rape survivors or those who are too emotional to discuss research participation.

Our initial recruitment process was dependent on the trained TCC staff's ability to identify eligible participants, introduce the study, gain consent to be contacted by us (the researchers) and finally collect accurate contact details. The project staff then contacted the potential participant, and invited them to our study clinic for formal consent procedures and the baseline interview (see Figure 1). Although TCC staff were keen to assist us, our study was not their priority. We therefore enlisted the Life Line counselling staff, who provide the initial counselling service at the rape centres and we contribute towards their salary. However, when funding (USAID funding) for counselling services at the rape centers decreased at the end of 2015, the RICE investigators decided to employ dedicated RICE recruiters because recruitment remained low and it made financial sense to employ staff to increase the enrollment rather than to enroll over a longer period. We employed two of the Life Line counselors to cover four recruitment sites. They set up the initial appointments, and arrange the collection and transportation of the participants to our research clinic. Frequently, participants become non-contactable after agreeing to attend our research clinic for formal consent procedures and this is a challenge. Participants provide incorrect contact numbers or our calls and voicemails remain unanswered. The latter often happens when final arrangements are being made or at time of transport pick-up. Such behaviour is also noted among the non-exposed group recruited at family planning clinics, and we suspect potential participants are reluctant to inform us directly that they do not want to participate in the research. They may feel socially coerced to agree, but find a way to discontinue contact with us. We know that rape survivors' lives are difficult post-rape and they change their phone numbers (phones are sometimes lost/stolen at the time of the rape), or they relocate. We also know that their mental health status interferes in their ability to regain normality.[13 74] This is an inherent challenge in our study, and staff are sensitive to rape survivors and accept that this group of women may not want to participant in our study.

Retention challenges and responses

Retaining participants in longitudinal studies is challenging and rape survivors bring additional concerns. Indeed, abused women have been identified as amongst the 'hard to reach' populations in research, similar to drug users.[38] Retention is also not often discussed in peer-review journal articles, where study findings are the focus. We searched for lessons learnt from other studies, but could not find longitudinal studies with large rape cohorts. Similarly, the HIV/AIDS sector has always grappled with retention in cohort studies.[46 75] As indicated above, we have participants who relocate to other provinces for safety reasons or as a coping strategy, or they find jobs in other towns. We are aware survivors experience a range of emotional difficulties with varying levels of distress and coping skills, and we know receiving support and care from others assist them on their road to recovery. We also know that there is poor integration of mental health within rape services, and although women can return to

For peer review only - http://bmjopen.bmj.com/site/about/guidelines.xhtml

the rape centre, they often choose not to. Our study has a full-time qualified trauma counsellor who provides counselling support at the study clinic. Many, however, do not use this facility and we may need to understand this better in follow-up studies. We find that women from both cohorts, who are counselled at our clinic, often seek assistance for their daily struggles. Women will report that their stresses are related to their difficult lives such as trying to find a job and securing income. Counselling sessions are often about assisting them to cope with these hardships. We expected more women to withdraw from the study because the research reminded them of the incident, but to date, among the 26 women who withdrew at the time of writing this paper, only two mentioned this as the reason. There may be others who have withdrawn, but because this has not yet been communicated to us, we consider them loss to follow up (LTFU).

We have activities beyond reimbursement to support retention. These include updating locator information at each visit, consent to use social media, maintaining telephonic contacts between clinic visits, individual birthday wishes via WhatsApp, offering transport from the rape centre, and a Saturday morning clinic to accommodate students and working women. We try to provide women with a pleasant experience at the clinic, and offer manicures and nail painting. We constantly explore other ways to improve their experience, and are currently exploring offering 10–15 minutes head and neck massages. At the end of 2015, our retention rate was between 50% and 75% (depending on the visits), and we intensified our retention activities through an incremental increase in reimbursement monies for each additional visit, a present (necklace/bag/earrings) for those who attend their 12- and 24-month visit and an additional snack of a small chocolate or a packet of peanuts. We employed a retention counsellor whose primary task is to track and monitor participant attendance, and to conduct problem-solving counselling of the retention barriers that are identified. We also employed a driver for home visits, initially for once a week, which then increased this to twice a week. In January 2017, we employed two drivers. One was dedicated to conduct daily home visits to those who missed a visit, and the other was employed to transport participants. However, home visits are not always successful. Sometimes, a home address is not found or the participant is not known by the household. This may be deliberate misinformation given to us as a form of avoidance. We also find cases where participants have left their rented homes and contact is completely lost. Nonetheless, many home visits result in finding participants or getting updated telephone numbers.

We developed an early warning system of missed attendance so that home visits are carried out soon after a missed appointment. These retention and tracking activities have become more complex because the number of baseline participants and follow-up visits increase. Each of our 2016 participants are expected to attend seven times (a total of 14 056 clinic visits). We started a monthly raffle where a grocery or pamper parcel worth R150.00 (US$10) can be won. Participants enter their study number into the visit box at each visit and there is a draw at the end of each month.

Transport to the clinic remains one of the key challenges. We recruit women from a wide geographic area and our study clinic within a regional hospital complex is only accessible by very unpredictable local taxi services. Participants report spending a long time travelling. Since the start of 2017, we have been offering all women transport to the clinic, and participants are collected in the morning at their homes, the rape centre or a known agreed landmark (police station/taxi rank etc.). Some women do not use the collection service because they profit slightly from the transport reimbursement and therefore prefer to use public transport. Transport coordination requires very good communication amongst staff members, and between the staff and participants. This is possible because almost all participants agree to use their personal phones to maintain contact with us and we commonly use Whatsapp – especially when arrangements are finalised after office hours.

For peer review only - http://bmjopen.bmj.com/site/about/guidelines.xhtml

Lessons learnt and limitations

We have learnt many lessons on how to set up and manage a longitudinal study with rape victims, and we continue to learn as our study progresses. We do not expect enrollment of rape-exposed participants to increase much beyond 26 per month because there are few other options left to increase recruitment from these four recruitment sites. Broadening the recruitment to other TCC sites is not financially feasible. We must accept that some women are not assertive enough to tell us that they do not want to participate in our study, and to avoid disapproval, they agree initially and thereafter evade contact. We must also be cognisant that power imbalances between researcher and participant may also be at play. More importantly, the post-rape period is very difficult for survivors and they have to negotiate many unsympathetic environments, which may include the rape services (health, police, justice and social), unsupportive family and friends, as well as a broader social environment wherein rape stigma remains a reality.[13 47]

Loss to follow-up (LTFU) is the most common cause of attrition in cohort studies[46] and the loss of contact with the participants has caused frustration among staff. Continuous support, and allowing staff to discuss their frustrations has enabled them to continue interacting sympathetically with trauma victims and to prevent vicarious trauma. The RICE study counsellor supports staff in addition to participants. We also ensure staff have access to an expert psychologist who spends a day with them every quarter to assist them in working with traumatised rape survivors. Staff have said they are encouraged when returning participants are clearly on a healing path (i.e. they are less depressed, or accept an HIV status and are starting ART), which makes the work a rewarding experience.

Difficulties in retention is well known in cohort studies and we have learnt much in this regard. In hindsight, we should have employed the retention counsellor earlier in the study and should have placed greater effort on the home visit programme. We are continuously assessing and discussing how to improve retention, and we hope the two recent initiatives (transport service and the raffle) will attract people to return. We may not be aware of all the factors influencing retention, and we hope the data from home visits and from our qualitative study will provide some insight to assist us in future longitudinal studies with sensitive participants. This study will also allow us to explore differential retention and loss to follow-up between the two groups. Mental health may also influence retention of women who are depressed and/or anxious and/or using substances, and are reluctant to attend the study clinic. The problem that women may struggle to attend care of any form after rape is well described in the literature on rape research because the attendance reminds them of the trauma.[76 77] This will affect overall retention.

We are aware of the limitation of generalising the findings to all rape survivors because low levels of reporting rape to the police is a well-known phenomenon locally and globally.[15 78] Women who report a rape are likely to be different in several aspects from the wider group of women who experience rape and who do report it. Those who do report are more likely to have experienced injuries, to have had witnesses, or to have been raped by non-partners (strangers). However, these are also women who are accessible from the wider population of women who are raped for longitudinal research on health outcomes. Women who come forward to the police or to health services are also the group who require services and so are an important group to study. From a pragmatic point of view, the methodology employed here is justifiable, especially considering logistical difficulty and the high cost of recruiting a population-based cohort. Generalisability may also be impacted on by the non-random, non-census nature of recruitment. Women may decline to participate in the study and this could introduce bias, but we expect this to happen in both groups. We will aim to reduce this bias, but will have to be cognisant that participation is by voluntary consent. It is also possible that some women

For peer review only - http://bmjopen.bmj.com/site/about/guidelines.xhtml

who decline participation may do so because of pre-existing mental health problems. However, our control group are also women who attend health services and, in this way, the two groups are similar.

ETHICS AND DISSEMINATION

Ethical approval for the study was granted by the South African Medical Research Council's Ethics Committee. Rape is a key element of our study, and the safety of participants is paramount and guides the research process. Project staff receive extensive training on the ethics, safety and protection of participants. We use lessons learnt from previous studies of participants recruited at rape centres.[47 48] We introduce the study to participants as a *Women's Health and Wellbeing Study* to protect women from being identified as rape survivors. The study follows the WHO ethical and safety guidelines on conducting research on gender-based violence and applies good clinical practice.[70] We also draw on guidelines developed for conducting intervention research on violence against women.[71] We provide women with sufficient information as required by the SAMRC's Ethics Committee on specific areas such as biometrics, children involvement (see below), genetic samples, HIV testing, hair samples and storage of blood. There is a separate consenting process for each of the above. We developed a brochure and information sheets for participants. Our previous experience highlighted the importance of providing full information, time to reflect and testing the understanding of consent before consent forms are signed. Participants are given a clear explanation of voluntariness of participation and the option of withdrawal is given at each visit.

Our study includes 16- and 17-year old girls who are recruited at one recruitment site (Crisis Centre). We did not receive approval from the NPA to recruit under-18s at the TCCs. For the children, we seek consent from both the parent/legal guardian and the child. It is important that the children we approach understand that their attendance at the rape centre to seek assistance will be revealed to the parent/legal guardian if disclosure has not yet occurred. We are cognisant that some girls may not want their parent/guardians to know about the rape event and we will not recruit girls if they are opposed to disclosure. The same applies to 16- and 17-year old children recruited at the family planning clinics. We consider 16-year olds to have sufficient maturity and mental capacity to understand the study (this is acknowledged in the Children's Act No. 38 of 2005 as amended by Act 41 of 2007), and the information sheet for them is the same as those for adult women. We only enrol a girl if both the parent/legal guardian and the child give consent to participate. We do not seek consent for HIV testing from the parent/legal guardian because according to South African law, parental consent is not required.

The research involves minimal risk to participants. However, questions may be intrusive and trigger emotions that can cause short-term psychological distress. All staff are trained to respond to any distress during the interviews and to provide containment. A trauma counsellor, employed at the start of the study, is available to provide support to participants and staff. Women who require further assistance are referred to appropriate services. The National Prosecuting Authority trained the staff on what may/may not be discussed with rape survivors to ensure the research does not compromise the legal process. To protect the project staff from having to testify in a trial and the integrity of the legal process, the questionnaire does not ask about circumstances of the rape until the rape case has been closed, or the trial has commenced. We expect to have many cases closed either by the survivors or by the investigating team by the end of the follow-up period as shown in research on rape attrition in South Africa.[72] We also do not have a concern for recall bias as such information on the outcome of the legal process is of great importance to most people.

We do not expect any major medical risks as part of the study. Participants may find the prospect of an HIV test distressing, but we anticipate that most participants would have been tested for HIV and would

For peer review only - http://bmjopen.bmj.com/site/about/guidelines.xhtml

know their HIV status from the rape care received, from antenatal care or from family planning services. In addition, there have been major national HIV testing campaigns across the country.[73] Our research nurses are trained to carry out HIV counselling and, together with the study trauma counsellor, can provide appropriate support and referral.

Project staff monitor adverse events at each visit. A serious adverse event is defined as experiencing violence by a partner due to participation in the study, or injury or hospitalisation due to the violence, or death of a study participant for any reason. The research nurse monitors and documents the occurrence of adverse events since the last study visit, at each visit. The nurse follows appropriate procedures for notification and responds to adverse events that arise. The number of events occurring in each cohort will be enumerated to determine whether there is a differential risk between study arms. The data manager will also monitor the adverse event data. Project staff are responsible for determining whether participation in the study is related to the adverse event or not, by exploring the circumstances surrounding the event. All events are reported to the SAMRC's Ethics Committee.

All staff are trained on matters of confidentiality and this is maintained in the retention activities. Contacting participants in the follow-up period poses a particular risk as identified in the Guidelines for conducting gender-based violence longitudinal studies,[71] and we only recruit participants who understand and agree on the follow-up nature of the study. At recruitment, we identify the different ways in which we can contact the participants (SMS, WhatsApp and home visits) and this is reviewed at each visit. All information is stored on secure computers or servers, and protected by a firewall and passwords. Study data is kept separate from the forms that can link data to the participants (consent forms and retention forms). Participants are compensated with R80.00 (US$5.50) at the baseline interview with an incremental addition of R20 (US$1.30) at each follow-up visit. Participants are compensated for travel costs or transport is arranged if required. Participants are offered snacks, such as a sandwich and a cool-drink, as well a choice of a small chocolate or a packet of nuts, during the visit to the research clinic.

Our study will generate an extensive set of results of interest to a wide audience. We will adopt a research uptake strategy that will involve policy makers, service providers, advocacy groups and academia, i.e. peer-review journal articles.

For peer review only

For peer review only - http://bmjopen.bmj.com/site/about/guidelines.xhtml

FIGURE LEGEND

Figure 1: RICE study flow chart of participants and samples

For peer review only

For peer review only - http://bmjopen.bmj.com/site/about/guidelines.xhtml

ACKNOWLEDGMENTS
We thank the South African National Department of Health, The KwaZulu-Natal Department of Health and the National Prosecuting Authority for assisting in accessing the participants. We also thank Life Line and Child Line for their assistance. We also thank the RICE research team for their commitment to the study and lastly, we thank all the participants for their time and sharing their lives with us.

For peer review only

For peer review only - http://bmjopen.bmj.com/site/about/guidelines.xhtml

COMPETING INTEREST DECLARATION

We have read and understood BMJ policy on declaration of interests and declare that we have no competing interests.

For peer review only

For peer review only - http://bmjopen.bmj.com/site/about/guidelines.xhtml

AUTHOR CONTRIBUTIONS

Naeemah Abrahams and Rachel Jewkes conceptualise the study and wrote the first draft of the proposal. Carl Lombard, Soraya Seedat, Andre Kengne, Gita Ramjee, Bronwyn Myers, Alesha Sewnath, Shibe Mhlongo, Nasheeta Peers and Claudia Garcia-Moreno contributed to the completion of the study protocol. Naeemah Abrahams wrote the first draft of this manuscript, and all authors contributed to and approved the final version to be published.

For peer review only

FUNDING
This research and the publication thereof is the result of funding provided by the South African Medical Research Council in terms of the SAMRC's Flagships Awards Project SAMRC-RFA-IFSP-01-2013/ RAPE COHORT.

For peer review only

For peer review only - http://bmjopen.bmj.com/site/about/guidelines.xhtml

REFERENCES

1. Devries K, Mak J, García-Moreno C, et al. The Global Prevalence of Intimate Partner Violence Against Women. Science 2013;**340**(6140):1527-28
2. World Health Organisation. Global and regional estimates of violence against women: prevalence and health effects of intimate partner violence and non-partner sexual violence. Geneva: World Health Organisation, 2013. http://www.who.int/reproductivehealth/publications/violence/9789241564625/en/
3. World Health Organisation. Post-Exposure Prophalaxsis to prevent HIV infection. Joint WHO/ILO guidelines on post-exposure prophylaxis (PEP) to prevent HIV infection: Geneva: WHO, 2007 http://apps.who.int/iris/bitstream/10665/43838/1/9789241596374_eng.pdf
4. Jewkes R, Dunkle K, Nduna M, et al. Intimate partner violence, relationship gender power inequity, and incidence of HIV infection in young women in South Africa: a cohort study. The Lancet 2010;**367**:41-48
5. Wood K, Maforah F, Jewkes R. 'He forced me to love him': Putting violence on adolescent sexual health agendas. Social Science and Medicine 1998;**47**(2):233-42
6. Pettifor AE, Kleinschimdt I, Levin JB, et al. A community-based study to examine the effect of a youth HIV prevention intervention on young people aged 15-24 in South Africa: results of the baseline survey. Tropical Medicine & International Health 2005;**10**(10):971-80
7. Dunkle K, Jewkes R, Brown HC, et al. Transactional sex among women in Soweto, South Africa: prevalence, risk factors and association with HIV infection. Social Science & Medicine 2004;**59**:1581-92
8. Jewkes R, Dunkle K, Nduna M, et al. Transactional sex and HIV incidence in a cohort of young women in the Stepping Stones trial. Journal of AIDS & Clinical Research 2012;**3**:158 doi: doi:10.4172/2155-6113.1000158[published Online First: Epub Date]|.
9. Jewkes R, Sikweyiya Y, Morrell R, et al. The relationship between intimate partner violence, rape and HIV amongst South African men: A cross-sectional study. PLoS One 2011;**6**(9)
10. Decker MR, Seage GR 3rd, Hemenway D, et al. Intimate partner violence functions as both a risk marker and risk factor for women's HIV infection: findings from Indian husband-wife dyads. J Acquir Immune Defic Syndr 2009;**51**:593-600
11. Dunkle KL, Jewkes RK, Nduna M, et al. Perpetration of partner violence and HIV risk behaviour among young men in the rural Eastern Cape, South Africa [. Aids. 2006 Oct 24;20(16):2107-14.
12. Jewkes R, Dunkle K, Koss MP, et al. Rape perpetration by young, rural South African men: Prevalence, patterns and risk factors. Soc Sci Med 2006;**63**(11):2949-61 doi: 10.1016/j.socscimed.2006.07.027[published Online First: Epub Date]|.
13. Abrahams N, R J, Mathews S. Depressive symptoms after a sexual assault among women, understanding victim-perpetrator relationship and the role of social percpetions. African Journal of Psychaitry 2013;**16**(4):288-93
14. Hembree EA, Street GP, Riggs DS, et al. Do assault-related variables predict response to cognitive behavioral treatment for PTSD? J Consult Clin Psychol 2004;**72**(3):531-4 doi: 10.1037/0022-006x.72.3.531[published Online First: Epub Date]|.
15. Jewkes R, Abrahams N. The epidemiology of rape and sexual coercion in South Africa: an overview. Social Science & Medicine 2002;**55**:153-66
16. Ehlert U, Gaab J, Heinrichs M. Psychoneuroendocrinological contributions to the etiology of depression, posttraumatic stress disorder, and stress-related bodily disorders: the role of the hypothalamus–pituitary–adrenal axis. Biological psychology 2001;**57**(1):141-52
17. Altemus M, Cloitre M, Dhabhar FS. Enhanced cellular immune response in women with PTSD related to childhood abuse. American Journal of Psychiatry 2003 Sep 1;160(9):1705-7

For peer review only - http://bmjopen.bmj.com/site/about/guidelines.xhtml

18. Bomyea J, Risbrough V, Lang AJ. A consideration of select pre-trauma factors as key vulnerabilities in PTSD. Clin Psychol Rev 2012;**32**(7):630-41
19. Prathikanti S, Weinberger DR. Psychiatric genetics – the new era: genetic research and some clinical implications. British Medical Bulletin 2005;**73-74**(1):107-22 doi: 10.1093/bmb/ldh055[published Online First: Epub Date]|.
20. Elklit A, Christiansen DM. ASD and PTSD in Rape Victims. Journal of Interpersonal Violence 2010 doi: 10.1177/0886260509354587[published Online First: Epub Date]|.
21. Zinzow HM, Resnick HS, McCauley JL, et al. The role of rape tactics in risk for posttraumatic stress disorder and major depression: Results from a national sample of college women. Depress Anxiety 2010;**27**(8):708-15
22. Rakyan VK, Down TA, Balding DJ, et al. Epigenome-wide association studies for common human diseases. Nature Reviews Genetics 2011;**12**(8):529-41
23. Coker AL, Davis KE, Arias I, et al. Physical and mental health effects of intimate partner violence for men and women. Am J Prev Med 2002;**23**(4):260-68 doi: http://dx.doi.org/10.1016/S0749-3797(02)00514-7[published Online First: Epub Date]|.
24. Keeshin BR, Cronholm PF, Strawn JR. Physiologic changes associated with violence and abuse exposure: an examination of related medical conditions. Trauma, Violence, & Abuse 2012;**13**(1):41-56
25. Logan JG, Barksdale DJ. Allostasis and allostatic load: expanding the discourse on stress and cardiovascular disease. J Clin Nurs 2008;**17**(7b):201-08
26. Golding JM. Intimate partner violence as a risk factor for mental disorders: A meta-analysis. Journal of family violence 1999;**14**(2):99-132
27. Rosengren A, Hawken S, Ounpuu S, et al. Association of psychosocial risk factors with risk of acute myocardial infarction in 11119 cases and 13648 controls from 52 countries (the INTERHEART study): case-control study. Lancet 2004;**364**(9438):953-62 doi: 10.1016/S0140-6736(04)17019-0[published Online First: Epub Date]|.
28. Rozanski A, Blumenthal JA, Kaplan J. Impact of psychological factors on the pathogenesis of cardiovascular disease and implications for therapy. Circulation 1999;**99**(16):2192-217
29. Das S, O'Keefe JH. Behavioral cardiology: recognizing and addressing the profound impact of psychosocial stress on cardiovascular health. Current hypertension reports 2008;**10**(5):374-81
30. UNAIDS. *Report on the Global AIDS epidemic*. Geneva: UNAIDS, 2012. http://files.unaids.org/en/media/unaids/contentassets/documents/epidemiology/2012/gr2012/20121120_UNAIDS_Global_Report_2012_with_annexes_en.pdf
31. Pettifor A, MacPhail C, Suchindran S, et al. Factors associated with HIV testing among public sector clinic attendees in Johannesburg, South Africa. AIDS Behav 2010;**14**(4):913-21 doi: 10.1007/s10461-008-9462-5[published Online First: Epub Date]|.
32. Chi BH, Yiannoutsos CT, Westfall AO, et al. Universal definition of loss to follow-up in HIV treatment programs: a statistical analysis of 111 facilities in Africa, Asia, and Latin America. PLoS Med 2011;**8**(10):e1001111 doi: 10.1371/journal.pmed.1001111[published Online First: Epub Date]|.
33. Nachega JB, Mutamba B, Basangwa D, et al. Severe mental illness at ART initiation is associated with worse retention in care among HIV-infected Ugandan adults. Tropical Medicine & International Health 2012;**Nov 8. doi: 10.1111/tmi.12019**
34. Cornell M, Schomaker M, Garone DB, et al. Gender differences in survival among adult patients starting antiretroviral therapy in South Africa: a multicentre cohort study. PLoS Med 2012;**9**(9):e1001304 doi: 10.1371/journal.pmed.1001304[published Online First: Epub Date]|.
35. Rothbaum BO, Foa EB, Riggs DS, et al. A prospective examination of post-traumatic stress disorder in rape victims. Journal of Traumatic Stress 1992;**5**(3):455-75 doi: 10.1007/bf00977239[published Online First: Epub Date]|.

For peer review only - http://bmjopen.bmj.com/site/about/guidelines.xhtml

36. Foa E. Cognitive behavioural treatments have long-term benefits in female rape survivors with post-traumatic stress disorder. Evid Based Ment Health 2012;**15**(4):89 doi: 10.1136/ebmental-2012-100764[published Online First: Epub Date]|.
37. Schnurr PP, Friedman MJ, Engel CC, et al. Cognitive behavioral therapy for posttraumatic stress disorder in women - A randomized controlled trial., Jama. 2007 Feb 28;297(8):820-30.
38. Gondolf EW, Deemer C. Phoning Logistics in a Longitudinal Follow-Up of Batterers and Their Partners. Journal of Interpersonal Violence 2004;**19**(7):747-65 doi: doi:10.1177/0886260504265618[published Online First: Epub Date]|.
39. Gondolf EW. Limitations of experimental evaluation of batterer programs. Trauma, Violence, & Abuse 2001;**2**(1):79-88
40. Machisa M, Jewkes R, Lowe Morna C, et al. The war @ home: Preliminary findings of the Gauteng Gender Violence Prevalence Study Gauteng: Gender Links, 2011. http://www.mrc.ac.za/gender/gbvthewar.pdf
41. UNAIDS. Global AIDS Response: Country Progress Report 2012, Republic of South Africa. Pretoria: Republic of South Africa, 2012. http://files.unaids.org/en/dataanalysis/knowyourresponse/countryprogressreports/2012countries/ce_ZA_Narrative_Report.pdf
42. Wand H, Ramjee G. Assessing and evaluating the combined impact of behavioural and biological risk factors for HIV seroconversion in a cohort of South African women. AIDS Care 2012;**24**(9):1155-62
43. Ramjee G, Coumi N, Dladla-Qwabe N, et al. Research Experiences in conducting multiple community-based HIV prevention trials among women in KwaZulu-Natal, South Africa. AIDS research and therapy 2010;**7**(10)
44. Criminal Law (Sexual Offences and Related Matters) Ammendment Act 32 of 2007. South Africa 2007. http://www.justice.gov.za/legislation/acts/2007-032.pdf
45. UNICEF. Thuthuzela Care Centres. Secondary Thuthuzela Care Centres 2012. http://www.unicef.org/southafrica/hiv_aids_998.html. .
46. Geng EH, Bangsberg DR, Musinguzi N, et al. Understanding Reasons for and Outcomes of Patients Lost to Follow-Up in Antiretroviral Therapy Programs in Africa Through a Sampling-Based Approach. Journal of acquired immune deficiency syndromes (1999) 2010;**53**(3):405-11 doi: 10.1097/QAI.0b013e3181b843f0[published Online First: Epub Date]|.
47. Abrahams N, Jewkes R. 'I believe I should not take the pills as people who are HIV positive take them': Barriers to Post Exposure Prophylaxis (PEP) Completion After Rape in South Africa. Cult Health Sex 2010;**12**:471-84
48. Abrahams N, Jewkes R, Lombard C, et al. Impact of telephonic psycho-social support on adherence to post exposure prophylaxis (PEP) after rape: a randomised controlled trial. AIDS Care 2010;**16**:1-9
49. Garcia-Moreno C, Jansen HAFM, Ellsberg M, et al. WHO Multi-country Study on Women's Health and Domestic Violence against Women: Initial results on prevalence, health outcomes and women's responses. Geneva, Switzerland: World Health Organisation, 2005. http://www.who.int/reproductivehealth/publications/violence/24159358X/en/
50. Jewkes R, Nduna M, Levin J, et al. A cluster randomised controlled trial to determine the effectiveness of Stepping Stones in preventing HIV infections and promoting safer sexual behaviour amongst youth in the rural Eastern Cape, South Africa: trial design, methods and baseline findings. Tropical Medicine and International Health 2006;**11**:3-16
51. Jewkes R, Christofides N, Vetten L, et al. Medico-legal findings, legal case progression, and outcomes in South African rape cases: retrospective review. PLoSMed 2009;**6**(10):e1000164

For peer review only - http://bmjopen.bmj.com/site/about/guidelines.xhtml

52. Vaux A, Phillips J, Holly L, et al. The Social Support Appraisals (SS-A) Scale: Studies of Reliability and Validity. American Journal of Community Psychiatry 1986;**14**(2):195-219
53. Jalbrzikowski M, Sugar CA, Zinberg J, et al. Coping styles of individuals at clinical high risk for developing psychosis. Early Interv Psychiatry 2012 doi: 10.1111/eip.12005[published Online First: Epub Date]|.
54. Singh D, Chaudoir SR, Escobar MC, et al. Stigma, burden, social support, and willingness to care among caregivers of PLWHA in home-based care in South Africa. AIDS Care 2011;**23**(7):839-45 doi: 10.1080/09540121.2010.542122[published Online First: Epub Date]|.
55. Sheehan DV, Sheehan KH, Shytle RD, et al. Reliability and validity of the Mini International Neuropsychiatric Interview for Children and Adolescents (MINI-KID). J Clin Psychiatry 2010;**71**(3):313-26 doi: 10.4088/JCP.09m05305whi[published Online First: Epub Date]|.
56. Nduna M, Jewkes R, Dunkle K, et al. Associations between depressive symptoms, sexual behaviour and relationship characteristics: a prospective cohort study of young women and men in the Eastern Cape, South Africa. J Int AIDS Soc 2010;**13**:44
57. Saunders JB, Aasland O, Babor T, et al. Development of the Alcohol Use Disorders identification test (AUDIT): WHO Collaborative project on early detection of persons with harmful alcohol consumption-II. Addiction 1993;**88**:791-804
58. Sinadinovic K, Wennberg P, Berman AH. Population screening of risky alcohol and drug use via Internet and Interactive Voice Response (IVR): a feasibility and psychometric study in a random sample. Drug and alcohol dependence 2011;**114**(1):55-60 doi: 10.1016/j.drugalcdep.2010.09.004[published Online First: Epub Date]|.
59. Vythilingum B, Roos A, Faure SC, et al. Risk factors for substance use in pregnant women in South Africa. S Afr Med J 2012;**102**(11):851-4
60. Gray MJ, Litz BT, Hsu JL, et al. Psychometric properties of the life events checklist. Assessment 2004;**11**(4):330-41 doi: 10.1177/1073191104269954[published Online First: Epub Date]|.
61. Davidson JR, Book SW, Colket JT, et al. Assessment of a new self-rating scale for post-traumatic stress disorder. Psychol Med 1997;**27**(1):153-60
62. Zlotnick C, Davidson J, Shea MT, et al. Validation of the Davidson Trauma Scale in a sample of survivors of childhood sexual abuse. J Nerv Ment Dis 1996;**184**(4):255-7
63. Bernstein D, Stein J, Newcomb M, et al. Development and validation of a brief screening version of the Childhood Trauma Questionnaire. Child Abuse & Neglect 2003;**27**:169-90
64. Jewkes R, Dunkle K, Nduna M, et al. Associations between childhood adversity and depression, substance abuse & HIV & HSV2 in rural South African youth. Child Abuse and Neglect 2010;**34**(11):833-41
65. Jewkes R, Levin J B, Penn-Kekana L. A. Gender inequalities, intimate partner violence and HIV preventive practices: findings of a South African cross-sectional study. Soc Sci Med 2003;**56**(1):125-34
66. Department of Health. The 1998 South African Demographic and Health Survey. Full Report. . Pretoria: Department of Health, 1999. https://dhsprogram.com/pubs/pdf/FR206/FR206.pdf
67. World Health Organisation. The WHO STEPwise approach to chronic disease risk factor surveillance (STEPS) (instrument v2.1). Geneva: World Health Organization. http://www.who.int/chp/steps/STEPS_Instrument_v2.1.pdf
68. World Health Organisation. Consolildated Guidelines on HIV testing services Geneva: World Health Organisation, 2015. http://www.who.int/hiv/pub/guidelines/hiv-testing-services/en/
69. Molenberghs G, Kenward M. *Missing Data in Clinical Studies*. Sussex England Wiley, 2007.
70. World Health Organization. *Putting Women First: Ethical and Safety Recommendations for Research on Domestic Violence Against Women*. Geneva, Switzerland: World Health Organization, 2001. http://www.who.int/gender/violence/womenfirtseng.pdf

For peer review only - http://bmjopen.bmj.com/site/about/guidelines.xhtml

71. World Health Organisation. Ethical and safety recommendations for intervention research on violence against women. Building on lessons from the WHO publication. Putting women first: ethical and safety recommendations for research on domestic violence against women. in World Health Organization, ed. Geneva, 2016. http://www.who.int/reproductivehealth/publications/violence/intervention-research-vaw/en/
72. Vetten L, Jewkes R, Fuller R, et al. Tracking Justice: The Attrition of Rape Cases Through the Criminal Justice System in Gauteng. Johannesburg: Tshwaranang Legal Advocacy Centre, 2008. https://www.csvr.org.za/docs/tracking_justice.pdf
73. Shishana O, Rehle T, Simbayi L, et al. South African HIV Prevalence, Incidence and Behavioural survey 2012. Cape Town Human Sceinces Research Council, 2014. http://www.hsrc.ac.za/uploads/pageContent/4565/SABSSM%20IV%20LEO%20final.pdf
74. Abrahams N, Gevers A. A rapid appraisal of the status of mental health support in post-rape care services in the Western Cape. 2017 2017;**23**(1) doi: 10.4102/sajpsychiatry.v23i1.959[published Online First: Epub Date]|.
75. Rosen S, Fox MP, Gill CJ. Patient Retention in Antiretroviral Therapy Programs in Sub-Saharan Africa: A Systematic Review. PLoS Medicine 2007;**4**(10):e298 doi: 10.1371/journal.pmed.0040298[published Online First: Epub Date]|.
76. Nesvold H, Friis S, Ormstad K. Sexual assault centers: attendance rates, and differences between early and late presenting cases. Acta Obstet Gynecol Scand 2008;**87**(7):707-15
77. Jones JS, Alexander C, Wynn BN, et al. Why women don't report sexual assault to the police: The influence of psychosocial variables and traumatic injury. The Journal of emergency medicine 2009;**36**(4):417-24
78. Kelly L, Lovett J, Regan L. *A gap or a chasm? Attrition in reported rape cases*. London: Home Office Research, Development and Statistics Directorate, 2005. https://www.researchgate.net/publication/238713283_

For peer review only

For peer review only - http://bmjopen.bmj.com/site/about/guidelines.xhtml

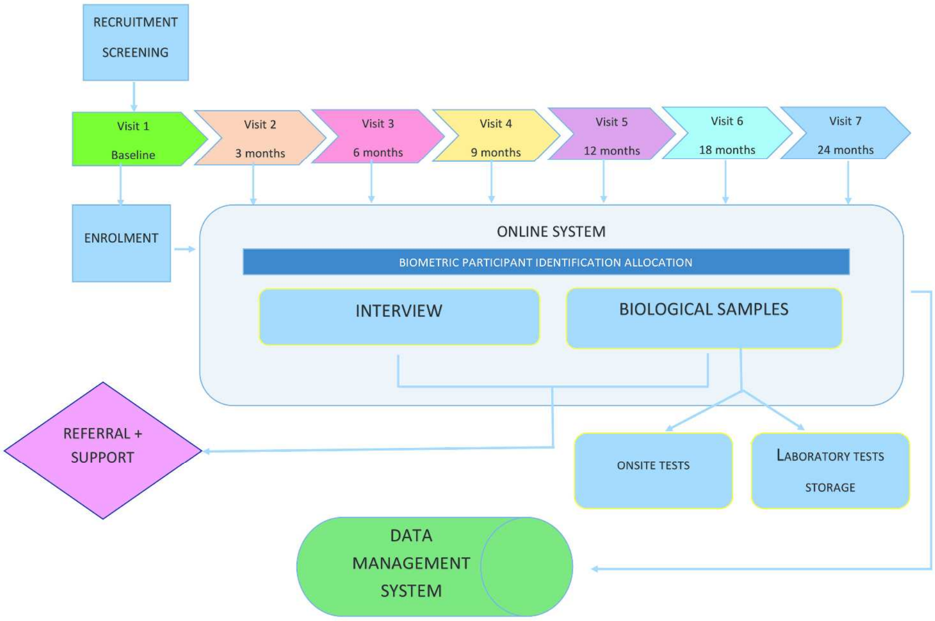

Figure 1: RICE study flow chart of participants and samples

297x209mm (300 x 300 DPI)
